# Non-Invasive Phenotyping Reveals Genomic Regions Involved in Pre-Anthesis Drought Tolerance and Recovery in Spring Barley

**DOI:** 10.3389/fpls.2019.01307

**Published:** 2019-10-25

**Authors:** Sidram Dhanagond, Guozheng Liu, Yusheng Zhao, Dijun Chen, Michele Grieco, Jochen Reif, Benjamin Kilian, Andreas Graner, Kerstin Neumann

**Affiliations:** ^1^Department of Genebank, Leibniz Institute of Plant Genetics and Crop Plant Research (IPK), Seeland, Germany; ^2^Department of Breeding Research, Leibniz Institute of Plant Genetics and Crop Plant Research (IPK), Seeland, Germany; ^3^BBCC – Innovation Center Gent, Gent Zwijnaarde, Belgium; ^4^Institute for Biology, Humboldt-Universität zu Berlin, Berlin, Germany; ^5^Plant Breeding Department, Martin-Luther-University Halle-Wittenberg, Halle, Germany; ^6^Global Crop Diversity Trust (GCDT), Bonn, Germany

**Keywords:** barley, biomass, drought stress, recovery, GWAS, growth, non-invasive

## Abstract

With ongoing climate change, drought events are becoming more frequent and will affect biomass formation when occurring during pre-flowering stages. We explored growth over time under such a drought scenario, *via* non-invasive imaging and revealed the underlying key genetic factors in spring barley. By comparing with well-watered conditions investigated in an earlier study and including information on timing, QTL could be classified as constitutive, drought or recovery-adaptive. Drought-adaptive QTL were found in the vicinity of genes involved in dehydration tolerance such as dehydrins (*Dhn4, Dhn7, Dhn8*, and *Dhn9*) and aquaporins (e.g. *HvPIP1;5, HvPIP2;7*, and *HvTIP2;1*). The influence of phenology on biomass formation increased under drought. Accordingly, the main QTL during recovery was the region of *HvPPD-H1*. The most important constitutive QTL for late biomass was located in the vicinity of *HvDIM*, while the main locus for seedling biomass was the *HvWAXY* region. The disappearance of QTL marked the genetic architecture of tiller number. The most important constitutive QTL was located on 6HS in the region of *1-FEH*. Stage and tolerance specific QTL might provide opportunities for genetic manipulation to stabilize biomass and tiller number under drought conditions and thereby also grain yield.

## Introduction

Barley breeding has not substantially changed total biomass (Austin et al., 1980; Gifford et al., 1984; Horie et al., 2005) but rather its distribution resulting in an increased harvest index (Abeledo et al., 2003; Reynolds et al., 1999). Consequently, one promising opportunity to increase grain yield in the future is to boost biomass *per se* (Fischer and Edmeades, 2010). Annual cereal yield increases in the European Union have fallen below 1% since the turn of the century (Noleppa, 2016) partly explained by an increasing volatility due to climate change (Brisson et al., 2010), leading to a higher frequency of drought periods (Lloyd-Hughes and Saunders, 2002; Lehner et al., 2006), affecting plant growth and causing yield losses world-wide (Jones and Corlett, 1992; Boyer and Westgate, 2004). Spring drought events reduce mainly vegetative biomass formation. Water deficit affects cell growth by hampering mitosis, cell elongation, and expansion (Farooq et al., 2009) and thereby reducing leaf area and tillering. Ongoing drought leads to a reduction in photosynthesis (Jedmowski et al., 2014). All these factors result in a reduced dry matter production with negative effects on grain yield. Though barley is well adapted to a wide range of climatic conditions (Ceccarelli and Grando, 1996), improvement of yield under drought environments has been challenging for plant breeders (Richards et al., 2002) as the effect of drought is highly depending on the time of onset, duration, and stress intensity. Despite that, numerous QTL studies in bi-parental mapping or natural collections have been conducted to identify genetic components of different drought stress tolerance in barley (e.g. Teulat et al., 2001; Forster et al., 2004; Guo et al., 2008; Mehravaran et al., 2014; Fan et al., 2015; Abou-Elwafa, 2016). There is evidence that selection for individual traits contributing to drought tolerance can improve grain yield (Edmeades et al., 1999; Richards, 2006).

Nevertheless, entirely different genetic loci were detected at different developmental stages within the same mapping population (Szira et al., 2008). Consequently, studying the genetics of drought tolerance requires phenotyping throughout the life cycle of a plant, nearly impossible to realize in the field. New phenotyping platforms that employ non-invasive imaging techniques (Berger et al., 2010; Chen et al., 2014a) in controlled environments offer the most suitable way to perform such experiments. Several studies demonstrate the suitability of non-invasive phenotyping for abiotic stress tolerance (Rajendran et al., 2009; Chen et al., 2014b; Honsdorf et al., 2014; Neumann et al., 2015). Recently, the genetic architecture of vegetative biomass formation was revealed in spring barley throughout plant development under well-watered conditions (Neumann et al., 2017).

Growth and development are regulated by plant hormones, flowering time genes, plant architecture genes, and many transcription factors. The main plant hormones are abscisic acid (ABA), indole-3-acetic acid (IAA or auxin), brassinosteroids (BRs), cytokinin (CK), gibberellic acid (GA), ethylene, jasmonic acid (JA), and salicylic acid (Santher et al., 2009). Therefore, genes regulating hormone levels or genes being activated or turned off by hormones can be candidates for growth-related traits. Auxin response factors (ARFs) regulate gene expression and have distinct roles in plant development, including root growth and leaf expansion, and are also involved in stress adaptation (Li et al., 2016). Recently, twenty different ARFs were identified in barley (Tombuloglu, 2019). The gene *HvDIM* is involved in BR biosynthesis, and mutations resulted in deficiency or reduced levels of castasterone, suggested end product of brassinosteroid biosynthesis pathway in cereal grasses (Kim et al., 2008), which generally results in reduced plant stature and sturdiness. Further, in rice, three *Short Vegetative Phase (SVP)*-like MADS-box genes which regulate meristem identity and flowering time (Trevaskis et al., 2007) were demonstrated to act as negative regulators of BR responses (Lee et al., 2008). The GA oxidases (GAoxs) are essential for the biosynthesis of bioactive GAs and cluster into eight subfamilies, from which GA2ox, GA20ox, and GA3ox have been well studied while the function of GAox-A/B/C/D genes has to be still explored (Huang et al., 2015). GA is not only a key regulator of growth and development (Yamaguchi, 2008) but also plays a role under abiotic stress by inducing growth restriction (Colebrook et al., 2014). CK plays a major role in vegetative development such as root and shoot meristem maintenance and root elongation and root branching (Osugi and Sakakibara, 2015) and contributes to drought tolerance by various mechanisms including water balance regulation (Pavlů et al., 2018). CK oxidases/dehydrogenases (CKX) inactivate the hormone in a single enzymatic step and are therefore controlling local CK levels (Schmülling et al., 2003). Potassium transporters are involved in the response to osmotic stress (Osakabe et al., 2013) as potassium is an essential micronutrient for plant growth and involved in enzyme activation, osmoregulation and further stomatal movement (Han et al., 2016). Fructan exohydrolases (FEHs) mobilize fructans that are important storage carbohydrates produced from sucrose (Van Riet et al., 2006) and thereby involved in plant growth. They are continuously accumulated during stem growth (Zhang et al., 2008). Moreover, fructans can stabilize membranes during drying and thereby help to prevent leakage during drought (Livingston et al., 2009).

Flowering time genes affect plant growth directly or by pleiotropy. Recently, the importance of *HvPPD-H1* on leaf growth was demonstrated, with insensitive types possessing a longer leaf growth duration and a higher cell number (Digel et al., 2016), most likely as a result of source-sink allocation. Moreover, flowering time gene expression is affected by abiotic stress, and increased for *HvPpd-H1*, *HvPRR73* and *HvPRR95* (Habte et al., 2014).

The plant-specific DOF transcription factors (Moreno-Risueno et al., 2007) have a suggested role in the regulation of important processes vital for plant development including the modulation of response to abiotic stress (Noguero et al., 2013), and shoot branching in *Arabidopsis* (Zou et al., 2013) as well as regulation of stomatal development (Negi et al., 2014). Recently, a rich diversity in *HvDof* genes was seen in a screen of 58 barley accessions (Rouhian et al., 2017). Further, BZIP transcription factors regulate many plant processes including stress signaling in *Arabidopsis* (Jakoby et al., 2002). In rice, *OsABF1* was induced by abiotic stresses and was connected with enhanced drought tolerance (Hossain et al., 2010).

Several genes are known that influence the water balance of plants. Aquaporins are water channels that facilitate water uptake in barley roots (Knipfer et al., 2011). The subfamily of plasma membrane intrinsic proteins (PIPs) has a crucial role for the water balance of plants (Hove et al., 2015; Afzal et al., 2016). The subfamily of tonoplast intrinsic proteins (TIPs) in the vacuolar membrane takes a supposed role in vacuolation, and expression of TIP genes is influenced by plant hormones GA and ABA in an antagonistic way (Lee et al., 2015). Dehydrins are LEA group 2 proteins upregulated in reaction to abiotic stresses such as cold, drought or salt stress, acting as hydration buffers and thereby reducing water loss (Graether and Boddington, 2014; Banerjee and Roychoudhury, 2016). Pectin methylesterases (PMEs) are deesterising pectin, the main cell wall component and thereby are involved in cell wall elasticity and porosity, crucial prerequisites for cell elongation and water uptake (Müller et al., 2013). Accordingly, PMEIs have a role in osmotic stress (Hong et al., 2010; Wormit and Usadel, 2018).

In the current study, we investigated the dynamics of the genetic architecture of biomass under a spring drought event by adapting our previously developed experimental setup (Neumann et al., 2015). In particular, we aimed at elucidating constitutive biomass QTL (stable under different environmental conditions) and adaptive biomass QTL (only representative for specific environmental conditions such as water-deficit conditions) by comparison to well-watered conditions investigated earlier in three consecutive experiments (Neumann et al., 2017).

## Material and Methods

### Spring Barley Mapping Panel and Experimental Set-Up

A set of 100 diverse two-rowed spring barley accessions, described by Neumann et al. (2017) where the 100 barley accessions were evaluated for growth under control conditions, was used in this study for the genetic investigation of biomass development under the influence of seasonal drought stress. Ninety-seven of these are the subset of a world-wide spring barley collection used in several other studies (e.g., Haseneyer et al., 2010; Pasam et al., 2012; Alqudah et al., 2014). The accessions were mainly originating from Europe and were selected based on a low variation in flowering time under field conditions (Pasam et al., 2012). For more details and the list of genotypes see Neumann et al. (2017).

The plants were grown in a greenhouse at IPK Gatersleben (51°49′23″ N, 11°17′13″ E, 112 m a.s.l.), equipped with the imaging-based high-throughput phenotyping system LemnaTec-Scanalyzer 3D system (LemnaTec GmbH, Aachen, Germany). Three consecutive independent experiments were performed from March 2013 to September 2013 (**Supplementary Table S1**) each with five replicates per genotype. Every experiment lasted for 58 days. Initially, two seeds per pot were sown and 7–9 days after sowing (DAS), depending on the experiment, plants were thinned-out to retain only one seedling per pot. Automated watering, imaging, and randomization were performed daily. The pots were daily watered to reach a fraction of (plant) available water (fAW) of 89% from DAS 1 to DAS 26 and from DAS 46 to DAS 58. Drought stress was imposed from DAS 27 until DAS 45 by watering to a target weight corresponding to 10% fAW (Supplementary Material, **Supplementary Figure S1**). Re-watering was performed with an absolute volume of 300 ml on DAS 45, and from DAS 46 on all plants received watering to 89% fAW. Note that images were taken before watering as plants first move through the imaging chambers. Therefore, the stress period lasted from DAS 27 to 45. Greenhouse conditions were set to 15 h light, 18/16°C day/night as in Neumann et al. (2017). Details of pot size, soil, and FC determination were as described by Neumann et al. (2015).

### Trait Evaluation From Images and Manual Evaluation

For details on image capturing see **Supplementary Material**. The image analysis was done employing the barley analysis pipeline in IAP (Klukas et al., 2014). A pixel volume termed digital biomass (DB, with *voxel* as a unit) was calculated as in Neumann et al. (2017). Biomass growth patterns in stress conditions could be bifurcated into two parts describing the stress period and the recovery phase (Chen et al., 2014b, see **Supplementary Material**). In the morning of DAS 59, above-ground biomass was harvested and measured as fresh weight (FW). Tillers (TN) were manually counted at DAS 27, 45, and 58, marking the start and end of the stress treatment as well as the end of the experiment. The images of all plants were visually inspected if plants reached the growth stage of tipping BBCH 49 (Witzenberger et al., 1989) within the imaging period. In contrast to the experiments for well-watered conditions, only in experiment 2 the majority of plants (432) reached BBCH 49. In experiments 1 and 3, only 224 and 255 out of 500 plants reached BBCH 49, respectively. Therefore, we could not further analyze this parameter.

### Phenotypic Analysis

Statistical analysis was performed in R software (R 2010). As plants were fully randomized each night, we considered the experimental design as a completely randomized design for statistical analysis. An outlier test was performed on the raw data from each of the three experiments. The outlier test was performed according to Tukey’s method (Anscombe and Tukey, 1963). Best linear unbiased estimates (BLUEs) were calculated for each day, within each experiment with the model *Y* = μ + *G* + *e* where *Y* is the vector of observed phenotypic value, µ is intercept, *G* is effect of genotype, e is residual for each plant, while µ and G were treated as fixed effects.

BLUEs from all 3 experiments were combined and used to detect outliers again and estimate BLUEs across experiments with the model *Y =* µ+ *G*+ *E* + *e*, where *Y* is the vector of BLUEs from a single experiment, µ is the intercept, *G* is the effect of genotype, *E* is the effect of experiments, *e* is the residual. While µ and *G* were treated as fixed effects, the other effects were treated as random. The BLUES across experiments were used to calculate the trait correlation. Moreover, we performed a one-step model to estimate the phenotypic variance components based on the raw data by fitting the model *Y = G + E + GxE+ e*, where *G* is the effect of genotype, *E* is the effect of experiments, *GxE* is the genotype by experiment interaction, and e is the residual, while assuming that all effects were random effects.

Broad-sense heritability was calculated as

$$H^{2}=\frac{V_{G}}{V_{G}+\frac{V_{\mathrm{GE}}}{O}+\frac{V_{e}}{\mathrm{OR}}},$$

where *V_G_*, *V_GE_*, and *V_e_* are the variance components of the genotype, genotype x experiment and the residual, respectively. *O* is the number of experiments for the respective DAS, and *R* the number of biological replicates.

### Genome-Wide Association Scans and Identification of Candidate Genes

The association mapping panel was genotyped using the 9k iSelect SNP array (Illumina, CA, United States). SNPs were filtered for missing data (5%) and minor allele frequency (MAF < 0.05), resulting in 4,866 polymorphic SNPs (DOI: 10.5447/IPK/2019/14) further used for genetic analysis with 4,122 SNPs having mapping information as described in Neumann et al. (2017). The decay of linkage disequilibrium (LD) and the structure of the association mapping panel were estimated by Neumann et al. (2017). The average LD decay amounted to 8 cM in the collection and a kinship using modified Rogers’ Distance (Reif et al., 2005) was sufficient to correct for population structure and included in the model for genome-wide association scans (GWAS). GWAS were performed using BLUEs from single experiments using the software ASReml-R 3.0 (Butler et al., 2009). The following mixed-linear model was applied as in Neumann et al. (2017):

Y=μ+E+S+G+e,

where μ is the overall mean, and *E* is the effect of the experiments, *S* is the effect of SNP, and *G* is the random effect of the genotype, while *e* is the residual error. This model considers a covariance structure of 2K $\sigma_{G}^{2}$ , where *K* refers to the kinship matrix (Jiang et al., 2015) and $\sigma_{G}^{2}$ is the genetic variance. A false discovery rate (FDR) with a significance level of 0.1 was applied for each trait and separately for all days (Benjamini and Hochberg, 1995). The proportion of explained genetic variance (GV) of the detected QTL was estimated as the adjusted r^2^ values standardized with the heritability.

We explored all loci for potential candidate genes using the recently annotated barley genome assembly (Mascher et al., 2017) using the gene sets from 2012 for genetic positions and 2016 for physical map positions (Colmsee et al., 2015). Designation of the *HvARF* genes was performed according to Tombuloglu (2019).

## Results

### High Heritability of Biomass Related Traits Under Drought Stress

The two-rowed spring barley panel was evaluated for tiller number and biomass over time in three experiments, which showed only minor seasonal differences (**Supplementary Table S2** and **Supplementary Figures S2** and **S3**). The high correlation between fresh weight and digital biomass justifies the use of digital biomass as a biomass proxy (**Supplementary Figure S4**). In addition, three stress-related traits were computed applying a biomass growth model: time A (TA) reflecting the time point when biomass development stopped, biomass at time A (DBA), and the re-growth rate (*k*) after re-watering started. Substantial variation for each trait was found in the collection (**Supplementary Table S3**).

Heritability of biomass over time was high (0.79 at DAS 12; 0.92 at DAS 58), although heritability decreased to 0.59 at DAS 45 beyond TA (**Supplementary Figure S5**). Also, all further traits showed a high heritability, reflecting the precision of the phenotypic data.

### Genotype-By-Time Interactions of Biomass Formation Are Modified by Drought Stress

**Figure 1:**
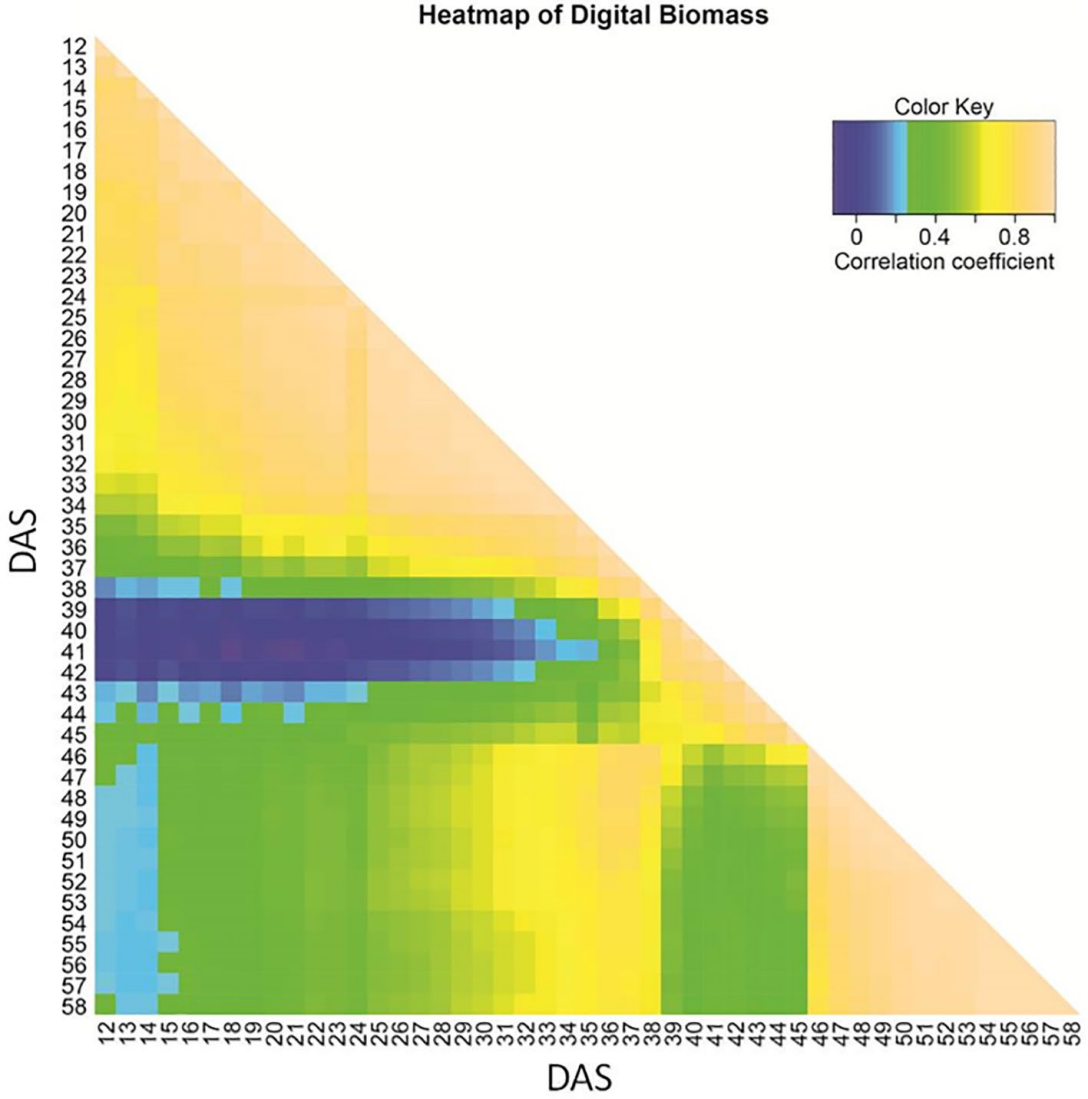
Heat map of Kendall correlation coefficient for image-based estimated biomass based on BLUEs across three experiments, shown from 12 to 58 days after sowing (DAS).

Correlation of biomass among different time points starting from DAS 12 until 58 varied (**Figure 1**). Early seedling and late vegetative biomass turned out to be unrelated. A moderate correlation (R ∼ 0.4–0.5) of biomass during progressing drought (DAS 27 to 37) to the final biomass (DAS 58) was observed. After the onset of wilting (time A), correlations to time points in other growth phases (early, recovery) declined. This pattern indicates a change in the genetic architecture of biomass formation during early and late drought phase and the recovery phase.

### Phenotypic Associations Between Biomass and Further Traits

Biomass and tiller number were correlated at all three investigated time points (**Supplementary Table S4**). Early tiller number possessed the highest correlation to biomass (R = 0.67), while late tiller number was less correlated (R = 0.42). Time A reflects the time point where growth has stopped; afterwards, (digital) biomass is decreasing due to a loss of turgidity, causing the wilting of leaves (Neumann et al., 2015). A highly negative correlation of time A with the biomass at the onset of the drought was observed; plants with higher initial biomass wilted earlier (**Supplementary Table S4**). The re-growth rate *k* was positively correlated with biomass and tiller number, indicating genotypes with higher biomass also have a higher capability to re-grow after a stress period. Further, we were interested at what fAW plants reached Time A, especially if there is a common water status where plant growth is arrested (transpiration > water extraction from the soil). Therefore, we calculated fAW at time A (fAW_TA) for each individual plant (see **Supplementary Material**). The parameter exhibited a high heritability of 0.68 suitable for GWAS. On average, genotypes stopped their growth at fAW_TA = 21%, but there was substantial variation in the panel (see **Supplementary Table S2**). Moreover, differences in fAW_TA were not related to a “biomass effect”, as no significant correlation to biomass at the onset of drought stress or biomass at time A was observed. Plants that are able to maintain growth even at lower fAW_TA maintained higher biomass in the last days of stress, resulting in a negative correlation of fAW_TA (not shown) and biomass (R ∼ −0.5) and reached Time A later (R = −0.52).

### Genetic Architecture of Biomass Under the Influence of Drought Stress

GWAS were performed for each time point. In total, 26 SNPs showed at least one significant association with digital biomass in the course of the experiment (**Supplementary Table S5**). These SNPs represent 12 different QTL and can be grouped according to their time of appearance. Six QTL were detected exclusively during the drought stress phase, five only during the recovery phase, while one QTL was associated with biomass in both phases. Before the onset of drought stress, no QTL was passing the FDR. Very similar GWAS results were obtained for digital biomass on DAS 58 and fresh weight on DAS 59 (**Supplementary Figure S6**). Only one significant SNP detected for fresh weight (6H, 24.5 cM) was not passing the FDR for digital biomass at any day despite –log(p) > 3 (**Supplementary Table S5**).

**Figure 2:**
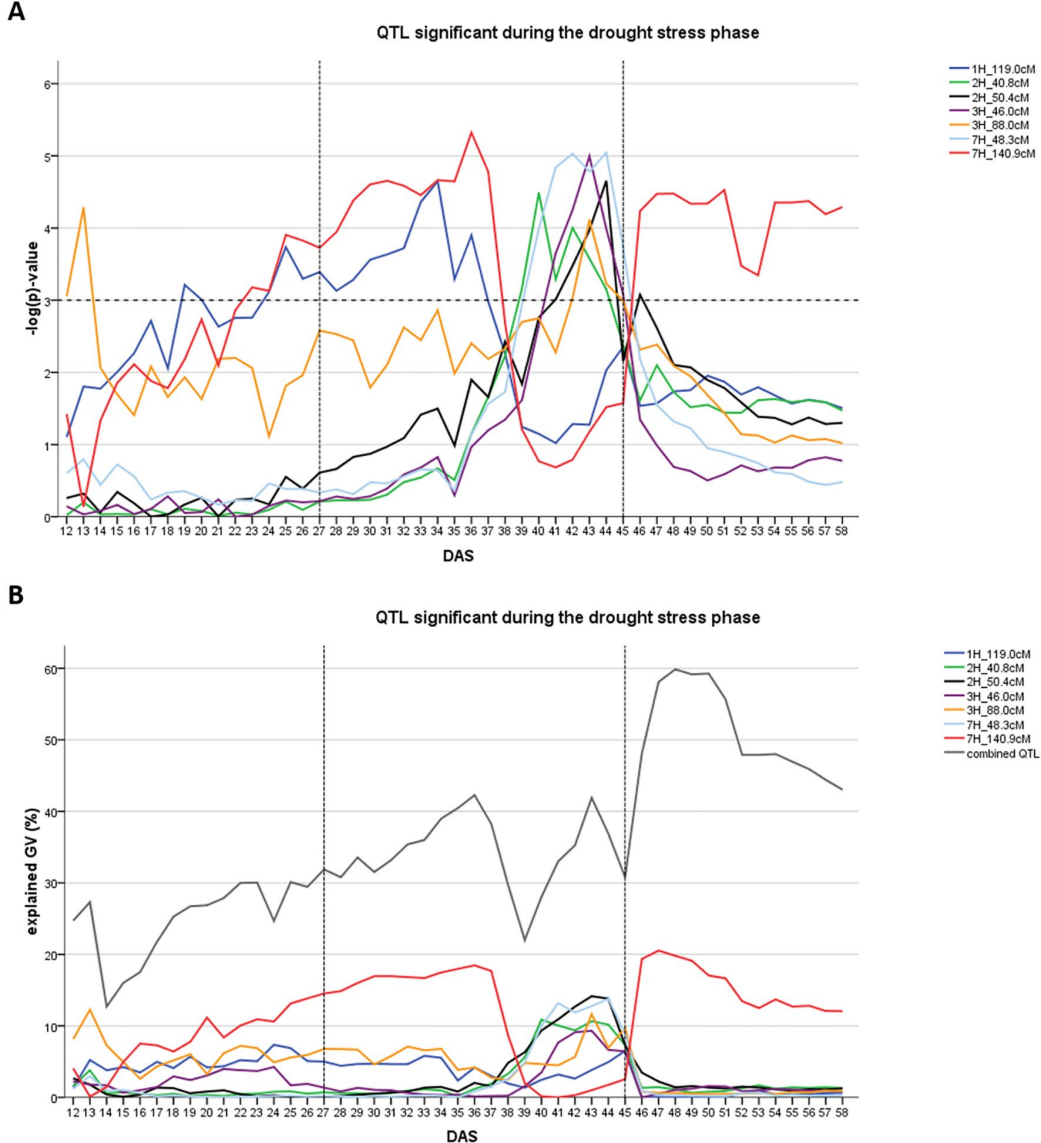
Time dynamics of QTL significantly associated (FDR < 0.1) with digital biomass (DB) during the drought stress phase in days after sowing (DAS). The drought stress phase from DAS 27 to DAS 45 is indicated by the two dashed vertical. **(A)** value –log(p) over time for each QTL (SNP with the highest significance) in different colors. Arbitrary threshold of significance at –log(p) = 3 is indicated by a dashed horizontal. **(B)** Proportion of genetic variance explained by each QTL, represented in different colors and combined (all 12 QTL detected during the whole time-course of the experiment – drought and recovery period).

The occurrence of QTL in the drought stress phase depended on the time of wilting (TA). Of the seven QTL, two were detected in the early drought phase before time A was reached (1H at 119.0 cM and 7H at 140.9 cM). The remaining five QTL were detected only after time A was passed (**Figure 2A**). The seven QTL together explained between 22.0 and 42.2% of the genetic variance during the time course of drought stress (**Figure 2B**). Individual QTL explained a maximum between 6.5 and 18.5% of genetic variance. The QTL on 7HL at 140.9 cM explained the highest amount of the genetic variance during the drought phase.

**Figure 3:**
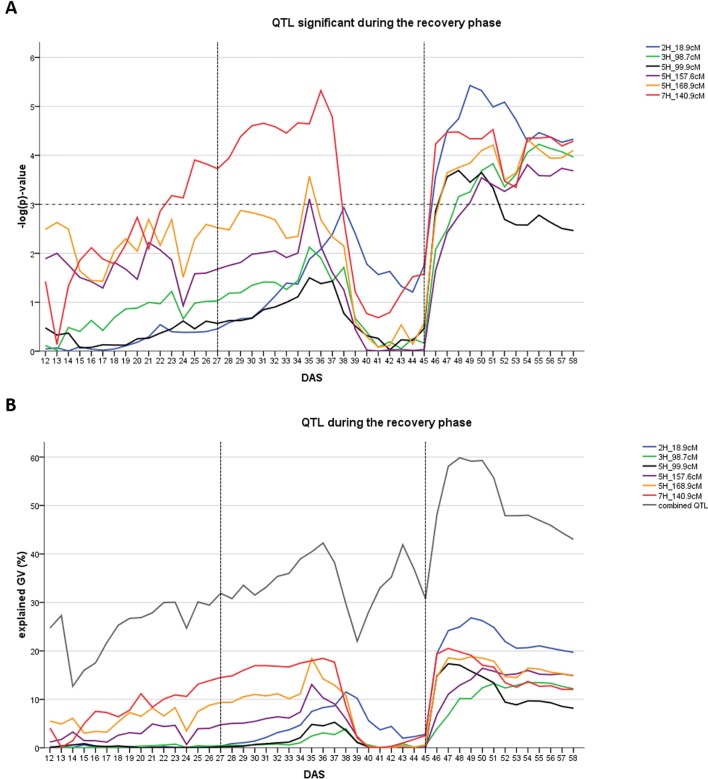
Time dynamics of association of QTL significantly associated (FDR < 0.1) with digital biomass (DB) during the recovery phase in days after sowing (DAS). The drought stress phase from DAS 27 to DAS 45 is indicated by the two dashed vertical lines. **(A)** Significance value –log(p) over time for each QTL (SNP with the highest significance) in different colors. Arbitrary threshold of significance at –log(p)=3 is indicated by a dashed horizontal line. **(B)** Proportion of genetic variance explained by each QTL, represented in different colors and combined (all 12 QTL detected during the whole time-course of the experiment – drought and recovery period).

In the recovery phase six different QTL were detected (**Figure 3A**). One of them was also present during the early drought stress phase (7H at 140.9 cM). One QTL was located in the region of the major flowering time locus *HvPPD-H1* (2H, 18.9–27.7 cM). Collectively, the six QTL explained between 43.0 and 59.9% of the genetic variance (Figure 3B). Individual QTL contribution was higher in the recovery phase compared to the drought phase: QTL explained between 13.5 and 28.8% of genetic variance with the *HvPPD-H1* locus being the most important QTL, followed by the QTL on 7H at 140.9 cM.

### Genetic Architecture of Novel Traits Obtained From Growth Curve Modelling

Four QTL were detected for Time A (**Supplementary Figure S7**, **Supplementary Table S5**): on 2H (135.8 cM), 6H (55 cM) and on 7H (14.0 and 140.9 cM). Here, the 7HL locus at 140.9 cM is represented by a different SNP (SCRI_RS_230261) compared to biomass (SCRI_RS_167617). The combined four QTL explain 40.2% of the genetic variance. Individually, the QTL on 7HS at 14 cM explained 22%, followed by those on 2HL (10.5%), 7HL (9.3%) and 6H (0.8%).

For biomass at time A (DBA), no SNP passed the FDR, although eight SNPs showed –log(p) values ≥ 3, including SCRI_RS_167617 on 7HL at 140.9 cM (**Supplementary Figure S7**).

The trait fAW_TA was significantly associated with three SNPs (**Supplementary Figure S8**, **Supplementary Table S5**) corresponding to one QTL on 3H (45.8–46.0 cM). The QTL explained 24.4% of the genetic variance.

For the re-growth rate *k* no SNP was passing the FDR, but 15 SNPs showed –log(p) > 3, including SCRI_RS_167617 on 7HL (140.9 cM) and two SNPs in the region of *HvPPD-H1* (**Supplementary Figure S9**, **Supplementary Table S5**).

### Genetic Architecture of Tiller Number Under the Influence of Drought Stress

Tiller number was counted at the start and at the end of drought stress as well as at the end of the experiments (DAS 27, 45 and 58, respectively). For each time point significant QTL were detected (**Supplementary Figure S10**, **Supplementary Table S5**).

In total, four QTL were significant for tiller number at DAS 27: 5H (43.7–45.7 cM), 5H at 120.1 cM, 6H at 30.1–30.2 cM and the biomass QTL on 7H at 140.9 cM. Together these four QTL explained 25.9% of the genetic variance, while the individual contribution ranged between 0.4% (5H 43.7–45.7 cM) and 20% (7H 140.9 cM).

For tiller number at DAS 45 three QTL were detected: 5H (43.7–45.7 cM), 6H at 30.1–30.2 cM, and 6H at 55 cM, the first two QTL identified already for tiller number at DAS 27. In combination, these three QTL explained 14.6% of the genetic variance. The most prominent QTL was the locus on 6H at ∼30 cM, which explained 18.6%, while the remaining two QTL explained only up to 0.64%.

For tiller number at DAS 58, three QTL were detected: 2H (74.1 cM), 5H (42.9–45.7 cM), and 6H at 30.1–30.2 cM. Together these explained 24.4% of the genetic variance. The most important QTL was on 6H at 30 cM, explaining 20.2% of genetic variance, while the QTL on 5H and 2H explained 4.5 and 2.2%, respectively.

## Discussion

### The Influence of Drought on Growth Patterns

The described experiments are complementary to the study of Neumann et al. (2017) where the same barley lines were evaluated under well-watered conditions (control treatment). The controlled conditions applied in the present study helped to eliminate small environmental effects that negatively impact heritabilities in drought stress experiments heritabilities (Rosielle and Hamblin, 1981; Fukai and Cooper, 1995; Kumar et al., 2007).

Comparing biomass under drought stressand control conditions, the ranking of genotypes changed during drought but quickly re-established once plants were re-watered (**Supplementary Figure S11**). At the start of stress treatment, plants had already more biomass and tillers compared to control treatment (**Supplementary Figures S12** and **S13**). The differences can be attributed to different seasonal scheduling of experiments (**Supplementary Table S6** compared with **Supplementary Table S1**). Therefore, future experimental series should be planned very carefully. Biomass was reduced in drought by 32% compared to control at the end of the experiment (**Supplementary Figure S14**). Directly upon re-watering, the reduction amounted to even 51% for biomass and 40% for tiller number. However, the re-tillering ability of barley (Jamieson et al., 1995) lead to an even slightly higher (although not significant) tiller number at the end of recovery phase compared to control treatment. Although the absolute number of tillers can be increased after drought (Blum et al., 1990), drought stress during the booting stage leads to a reduction in the number of productive tillers and a concomitant decrease in grain number and seed set (Lawlor et al., 1981; Rajala et al., 2010; Mathew et al., 2019).

Akin biomass, tipping time was affected by seasonal differences (see** Supplementary Material**). Vegetative drought stress can delay flowering as reported for wheat (Sanad et al., 2019) and rice (Kondhia et al., 2015; Haque et al., 2016). Early biomass was negatively correlated with tipping, but with progressing growth and development, the signs changed (**Supplementary Figures S15** and **S16**), only disrupted by the wilting of plants. Biomass in the recovery period was then even stronger correlated with tipping compared to the control treatment. Accordingly, the QTL in the *HvPPD-H1* region was the most important in the recovery phase whereas it was not significant under control conditions. Plant recovery is so far a mainly unexplored trait. However, a recent study in wheat showed that the ability to recover might be useful for selecting drought tolerant genotypes as visually scored plant recovery was correlated with grain yield under a vegetative drought set up in 16 diverse genotypes (Sanad et al., 2019).

### Genetic Architecture of Biomass Under the Influence of Drought Stress

By comparing the QTL identified in both treatments, it is possible to determine constitutive and adaptive QTL based on the definition given by Collins et al. (2008) who referred to a constitutive QTL when it is present in all environments whereas adaptive QTL are only present in a subset of environment(s). By this definition, we considered a QTL as constitutive when it was i) significantly associated with biomass in both treatments or ii) significantly associated in one treatment and having, –log(p)-values >3 in the complementary treatment (although missing the FDR threshold). Accordingly, we considered a QTL as adaptive if it was only in one treatment significantly associated and in the complementary had –log(p)-values <3.

The comparison of the results from this study with previously published QTL data benefitted in two ways: Firstly, our barley panel is part of larger collection consisting of 224 genotypes, which had been employed in a series of QTL studies, enabling a direct comparison of QTL (Pasam et al., 2012; Alqudah et al., 2014; Alqudah et al., 2016; Alqudah et al., 2018; Abdel-Ghani et al., 2019; Zhongtao et al., 2019). Secondly, comparison to other GWAS studies in barley benefitted from the widespread deployment of the 9K iSelect array (**Table 1**).

**Table 1:** Summary GWAS results from non-invasive phenotyping of growth under drought stress. All genomic regions significantly associated with one or more traits are presented along with the highest –log(p)-value of each trait (number of significant SNPS in brackets) along with other traits reported to be associated with these regions from the literature where iSelect marker platform was deployed.

Map position (cM)	Trait	Highest –log(p)	References of reported QTL in the QTL region
1H 119.0	DB 27–45 (1)	4.65	development^1^; malting quality – malt extract^2^; biomass yield (control) and osmotic adjustment^3^; root traits^21^
2H 18.9–27.7	DB 46–58 (11)	5.43	development^1^; tiller number^4^; grain yield^5^; flowering^6^; shoot elongation phase^7^; malting quality – malt extract^2^; heading date^8^; heading time^9,10^; biomass yield (DSI)^3^; seedling root and shoot traits^20,21^; leaf length and growth rate in control and salt stress^22^
2H 40.8	DB 27–45 (1)	4.49	development^1^; seedling shoot and root traits under osmotic stress^20^
2H 50.4	DB 27–45 (1)	4.66	tiller number (53.2 cM)^4^; malting quality, protein content^2^; biomass, osmolality and SPAD (stress)^3^; leaf blade area^11^; seedling root traits^21^
2H 74.1	TN58 (1)	4.07	development^1^; leaf blade area^11^; grain and spike number^12^; root Cl^–^ content^13^; malting quality^2^; TKW,protein content^9^; seedling root traits^20^
2H 135.8	TA (1)	4.79	leaf blade area^11^; biomass DSI (133.3 cM) and osmotic adjustment (135.8 cM)^3^; seedling root traits^20,21^; leaf length salt stress^22^
3H 45.8–46	DB 27–45 (3)	5.00	development^1^; leaf blade area (49.3cM)^11^; height (51.8 cM)^15^; biomass and SPAD (control) (51.2 cM) ^3^; seedling root traits^21^
	fAW_TA (3)	4.66	
3H 88	DB 27–45 (1)	4.28	tiller number^4^; leaf blade area^11^; shoot elongation, flowering^7^; malting quality^2^; biomass (control), osmolality (stress)^3^; leaf number^22^
3H 98.7	DB 46–58 (1)	4.72	development^1^; leaf blade area (96.5 cM)^11^; spike density^14^; grain yield^5^; shoot elongation phase, flowering^7^; malting quality, protein content^2^; heading^10^; biomass in control, DSI (100.3 – 106 cM) ^3^ and osmolality in stress^3^; root to shoot ratio^20^; growth rate in salt stress (104 cM)^22^
4H 91.0	DB 27–45* (1)	3.70	biomass, DSI^3^; seedling root traits^20^
5H 41.3–45.7	TN 27 (20)	3.44	development^1^; height and tiller number^4^; leaf blade area^11^; shoot elongation^7^; malting quality, protein content, malt extract^2^; heading^8^; protein content^15^; height^9^; biomass (control & stress), osmolality and SPAD (stress)^3^; seedling root traits^20^
	TN45 (22)	5.80	
	TN58 (25)	5.62	
5H 99.9	DB 46–58 (1)	3.69	development^1^; height^4^; shoot elongation^7^; kernel plumpness (95 cM)^15^; flowering^10^; osmolality (stress) (95 cM)^3^; shoot weight^19^
5H 120.1	TN27 (1)	3.47	tiller number (122.4 cM)^4^; leaf blade area (118.8 cM)^11^; malting quality, kernel plumpness^2^; flowering^10^
5H 139.1	DBA* (1)	3.90	osmotic adjustment (137.9 cM), biomass control and drought stress, DSI^3^; seedling root traits in osmotic stress^20^
5H 157.6	DB 46–58 (1)	3.81	malting quality, malt extract^2^; amylose content in grains (155.6 cM)^16^; shoot weight (154.2 cM)^21^
5H 169.4	DB 46–58 (1)	4.33	development^1^; shoot elongation^7^; malting quality, α-amylase^2^; biomass-DSI (167.9 cM)^3^; rhizosheat weight^19^; seedling root and shoot traits^20^; leaf length in salt stress^22^
6H 24.5	FW 59 (1)	3.69	tiller number^4^, biomass^3^
6H 30.1–30.2	TN27 (2)	3.59	leaf blade area^11^; β-glucan content in grains^16^
	TN45 (3)	5.80	
	TN58 (3)	5.62	
6H 55.0	TA (2)	4.15	height and tiller number^4^; leaf blade area^11^; TKW and spike number^12^; root Na^+^/K^+^ content^13^; malting quality, protein content^2^; height and TKW^9^; lodging^17^; shoot weight^19^; seedling root traits^20,21^; growth rate^22^
	TN45 (2)	3.53	
6H 105.1	DBA* (1)	3.19	biomass and DSI^3^
7H 14.0	TA (1)	4.30	straw yield^18^; development^1^; tiller number^4^; leaf blade area^11^; seedling root traits^20^; leaf number in salt stress^22^
7H 47.7–48.3	DB 27–45 (2)	5.04	leaf blade area^11^; tiller number^13^; malting quality, malt extract^2^; biomass (control, stress), DSI^3^; seedling root and shoot traits^20^
7H 140.9	DB 27–45 (1)	5.32	leaf blade area^11^, shoot weight^19^
	DB 46–58 (1)	4.67	
	TA (1)	4.21	
	TN27 (1)	3.62	

SNPs are described by their genetic position and associated traits. Identified QTL regions are compared to agronomic and growth/drought related traits from other mapping studies in barley that used the same barley collection and/or SNPs from iSelect or barley oligo pool array (BOPA), DB, digital biomass; FW, fresh weight; TN, tiller number (at a specific number of DAS); TA, Time A (stop of growth); DBA, DB at TA; fAW_TA, fraction of (plant) available water at TA.

*Not passing the FDR level. ^1^Alqudah et al. (2014), ^2^Mohammadi et al. (2015), ^3^Wehner et al. (2015), ^4^Alqudah et al. (2016): Note, only QTL for TN in two-rowed panel are included in the comparison. ^5^Ingvordsen et al. (2015), ^6^Maurer et al. (2015), ^7^Maurer et al. (2016), ^8^Muñoz-Amatriaín et al. (2014), ^9^Pasam et al. (2012), ^10^Sannemann et al. (2015), ^11^Alqudah et al. (2018), ^12^Gawenda et al. (2015), ^13^Long et al. (2013), ^14^Houston et al. (2013), ^15^Pauli et al. (2014), ^16^Shu and Rasmussen, (2014), ^17^Tondelli et al. (2013), ^18^Al-Abdallat et al. (2017), ^19^George et al. (2014), ^20^Abdel-Ghani et al. (2019), ^21^Zhongtao et al. (2019), ^22^Ward et al. (2019)

### Constitutive Biomass QTL

**Figure 4:**
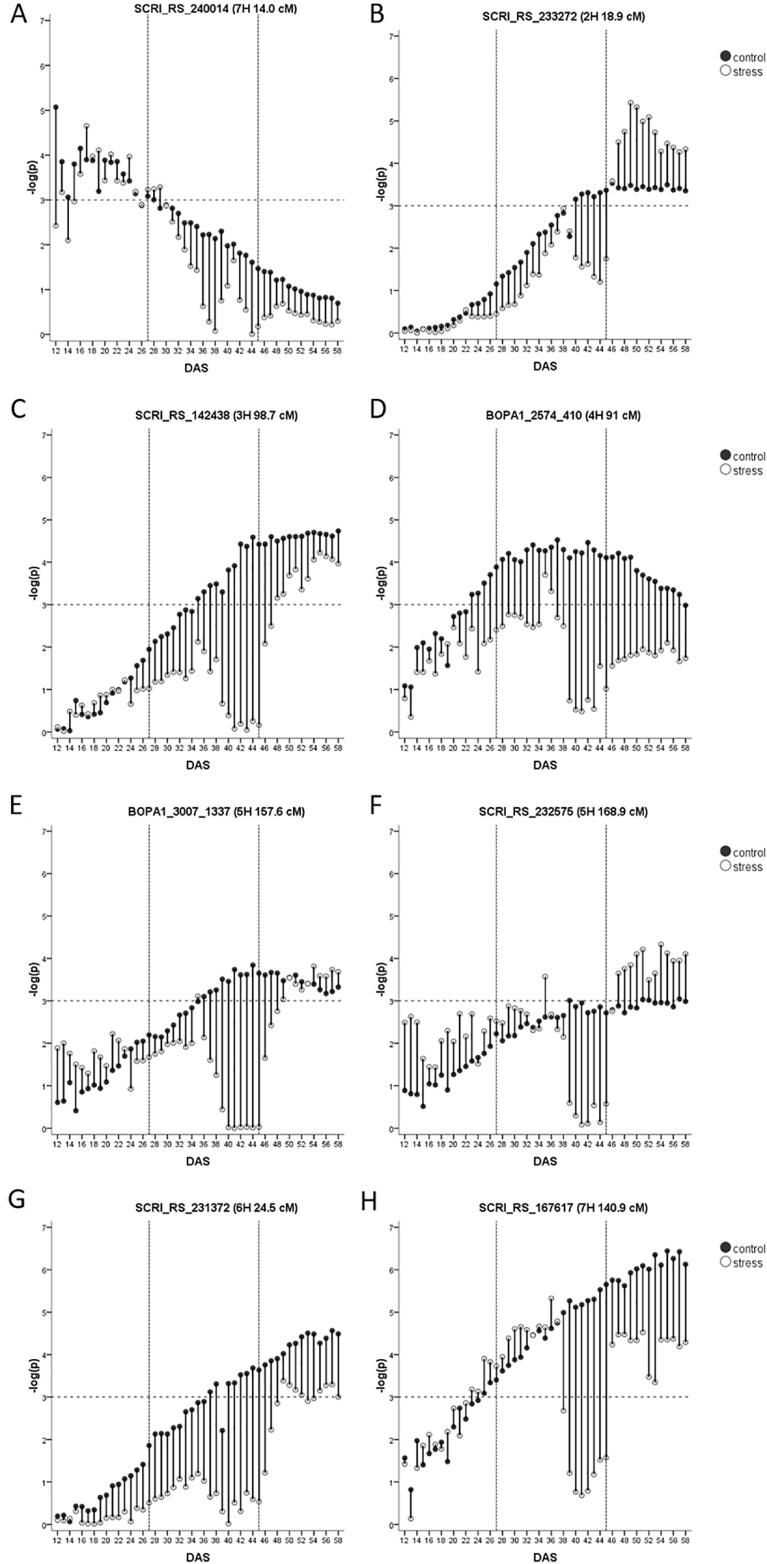
**(A**–**H)** Constitutive biomass QTL with their –log(p)-values over time in days after sowing (DAS) in drought stress (unfilled) and well-watered (black) conditions. Presented is only the most significant SNP of each QTL (in case it consists of several SNPs). As the FDR is calculated for each day and in each treatment separately, the general significance level of –log(p)-value = 3 is indicated by a dashed horizontal line. The drought stress phase from DAS 27 to DAS 45 is indicated by the two dashed vertical.

Across both studies, 17 biomass QTL were detected, eight of them are classified as constitutive. The first on 7HS at 14.0 cM located in the vicinity of *HvWAXY* is the only seedling biomass QTL. It had very similar –log(p)-values in both studies (**Figure 4A**) but did not pass the FDR in the current study. In the full barley panel, a QTL for root thickness at seedling stage was associated with the same SNP (SCRI_RS_240014) and QTL for tiller number, leaf blade area and tipping time were mapped to the same region (**Table 1**). A high level of vegetative biomass at early growth stages may translate to biomass at maturity in conditions with little or no rainfall after plant establishment. Accordingly, in a different barley panel, a QTL for final straw yield in Jordanian field conditions was detected here.

The most prominent QTL during the recovery phase was the *HvPPD-H1* locus. The QTL was not passing the FDR in control but already showed –log(p)-values >3 (**Figure 4B**). However, the role of this QTL has increased under the influence of drought, which is in line with increased expression levels of this gene under drought stress (Habte et al., 2014). The region was further detected as a hotspot for seedling growth in the full barley panel under normal and osmotic stress conditions, and co-locates with many agronomic traits (**Table 1**).

The QTL on 3HL at 98.7 cM (**Figure 4C**) passed the FDR in both studies and explained a very similar proportion of the genetic variance. In the full barley panel, the same SNP (SCRI_RS_142438) was associated with the root to shoot ratio under osmotic stress. Further, QTL for biomass, the drought susceptibility index (DSI) and osmotic adjustment in DS in a winter barley panel were located in close distance (**Table 1**). The QTL is located in the vicinity of flowering time gene *HvCMF1* (Cockram et al., 2012) and of AK366153, a supposed homolog of *LpABCG5* from *Lolium perenne* L. (**Supplementary Table S7**). This member of the ATP-binding cassette protein subfamily G was identified as a candidate behind a QTL for plant architecture (Shinozuka et al., 2011). In accordance with these two candidate genes, this genomic region is associated with many agronomic important traits such as heading, grain yield, or plant height (**Table 1**).

The constitutive QTL on 4HL at 91.0 cM was the only QTL found during the middle of the growth observation period under control conditions but did not pass the FDR under drought stress (**Figure 4D**). At close distance (90.9 cM) a QTL was detected for biomass and the DSI in winter barley, while in the full barley panel, root length QTL were located at 91.1 and 91.7 cM under control and osmotic stress conditions, respectively (**Table 1**). We detected three growth-related candidate genes in this region (**Supplementary Table S7**). The MADS-box transcription factor *BM1* is located in the physical map interval of the QTL. *Bm1* is one of the *SVP* genes and is mainly expressed in vegetative tissues such as nodes and internodes (Schmitz et al., 2000) and has a suggested role in vegetative growth (Carmona et al., 1998). Digel et al. (2016) observed a complete downregulation of *Bm1* upon floral transition. The expression pattern of *BM1* observed by Trevaskis et al. (2007) fits with the timing of our QTL. A second growth-related candidate gene is one ARF that is in close physical distance. Further, MLOC_17989.1 annotated to transport potassium is located at 91.4 cM and shows a high similarity to *HvHAK1* on 2H (58.7 cM). A drought-related candidate represents *HvTIP1;1* at 91.7 cM (**Supplementary Table S7**).

The QTL on 5HL at 157.6 cM was significant for biomass during the recovery phase of the drought treatment but did not pass the FDR under control conditions although timing and –log(p)-values were very similar (**Figure 4E**). In 3.5 cM distance, a QTL for seedling shoot weight was identified in the full barley panel (Zhongtao et al., 2019). *HvCesA2* resides at 158.3 cM, encoding a cellulose synthase expressed in seedling leaves and required for cell wall synthesis (Burton et al., 2004). Further, a potential homolog of the rice *OsFEN-1b* gene is located at 157.8 cM. It is encoding for a Flap endonuclease-1 (**Supplementary Table S7**) with a putative role in cell proliferation in the shoot apical meristem and young leaves Kimura et al. (2003).

Another constitutive QTL on 5HL at 169.4 cM was significant for biomass during the recovery phase but did not pass the FDR in the control treatment (**Figure 4F**). At the seedling stage, the region was associated with growth under non-stress and osmotic stress conditions in the full barley panel. In the same region, a constitutive QTL for biomass and for DSI and a QTL for rhizosheat were detected in other collections (**Table 1**). Interestingly, this genomic region harbors a key enzyme in the biosynthesis pathway of gibberellin, *HvGA20ox1*. In wheat, *TaGA20ox1* is expressed mainly in the nodes and ears of the elongating stem and in developing and germinating embryos (Appleford et al., 2006). The rice homolog *OsGA20ox1* has a role in seedling vigor and plant stature (Abe et al., 2012) as well as biomass (Oikawa et al., 2004). Drought-related candidates represent *HvPIP2;8* and the heat shock transcription factor *HvHsfA1a* (**Supplementary Table S7**). It is mainly known as a key factor of heat stress sensing in *Arabidopsis* (Liu et al., 2013); a role under drought in barley is so far unknown. However, in tomato, overexpression of *HsfA1a* was enhancing drought tolerance (Wang et al., 2015).

A constitutive biomass QTL is located on the short arm of 6HS at 24.5 cM (**Figure 4G**), although it failed the FDR threshold in drought treatment. At a very similar position (24.8 cM) a QTL for biomass in winter barley was identified (**Table 1**). Nearby, we detected a constitutive QTL for tiller number (30.1–30.2 cM), and it remains open if these are independent QTL or represent the same locus, especially as the SNP at 24.5 cM is not located on the physical map. There is no LD between SNPs of the two loci, indicating independence of the QTL. However, in the full barley panel, QTL for tiller number were identified at 24.5 cM and 28.5 cM along with a QTL for the leaf blade area at 28.5 cM (**Table 1**). Interestingly, *1-FEH* encoding a β-fructan 1-exohydrolase is located at 28.6 cM (Nagaraj et al., 2004). A further gene at 32.0 cM shows high similarity to *1-FEH* (**Supplementary Table S7**). In wheat, three homologs of the *1-FEH* gene were mapped to 6A, 6B, and 6D (Zhang et al., 2008). *1-FEh w3* (6B) conferring a higher TKW under drought conditions shows highest sequence similarity with barley *1-FEH* (Zhang et al., 2015), while *1-FEh w2* (6D) is involved in translocation of sugar from stem to grains (Yáñez et al., 2017).

The constitutive biomass QTL on 7HL at 140.9 cM (SCRI_RS_167617) was in both studies passing the FDR (**Figure 4H**). In control conditions the QTL started to be significant at DAS 33. By contrast,it started to disappear with progressing drought until plants were re-watered. Only the QTL at *HvPPD-H1* explained a higher proportion of the genetic variance in the recovery phase. Therefore, the 7HL locus represents the most stable and important biomass QTL in our barley panel. The same SNP was associated with seedling shoot weight in a different spring barley population (George et al., 2014). In the full barley panel, the region was revealed as a hotspot for sshoot and root traits at seedling stage (Abdel-Ghani et al., 2019), indicating the relevance of this locus also at earlier developmental stages. Very close to the QTL resides *HvDIM* and further genes involved in growth (**Supplementary Table S7**).

While most constitutive biomass QTL were co-locating with QTL for biomass in winter barley (Wehner et al., 2015), the QTL on 7HS and 7HL seem to be of importance only in spring barley. More research is needed to understand the role and timing of biomass QTL in the different genetic backgrounds (six-rowed barley, winter types) and field studies have to be carried out to verify their relevance and their relation to grain yield.

### Adaptive Biomass QTL

In total we classified nine biomass QTL across both studies as adaptive, seven of them were passing the FDR only in the current study for growth in drought conditions, while the remaining two QTL were detected only in the control treatment.

### Adaptive Biomass QTL for Biomass Prior to Drought or During Early Drought Phase

Two adaptive seedling biomass QTL, located at 3HL at 105.9 cM and 4H at 43.6 cM were detected only under well-watered conditions (**Supplementary Figures S17A, B**). The discrepancy in QTL detection for early biomass in both studies reflects the different seasonal scheduling, and a slightly shorter observation period in the drought study. In control, significant QTL for early biomass were detected between DAS 10 and 12, but in the current study, side view images needed for estimation of biomass were available only from DAS 12 onwards. In the full barley panel, both QTL regions were associated with root traits at seedling stage (Abdel-Ghani et al., 2019; Zhongtao et al., 2019), underlining their relevance for seedling growth. A strong candidate gene for the QTL on 3HL is *HvGA20ox3*, while for 4H the candidate might be a WRKY transcription factor (**Supplementary Table S7**). In *Arabidopsis* some of the WRKYs were shown to promote brassinosteroid regulated plant growth (Chen and Yin, 2017).

One adaptive QTL located on 1HL at 119 cM was significant at the beginning of drought treatment (**Supplementary Figure S17C**). However, we obtained –log(p) > 3 already before the onset of drought. Agronomic and drought-related QTL were mapped to the same region in the full barley panel or different collections (**Table 1**). *HvARF20*, and a gene with high similarity with *TaGA2ox8* reside very close to this QTL (**Supplementary Table S7**).

Another adaptive QTL on 3HL at 88 cM showed high –log(p)-values already prior drought but only turned significant at DAS 43 (**Supplementary Figure S17D**). It co-locates with a QTL for tiller number and leaf blade area in the full barley panel and with a QTL for biomass, DSI, and osmolality in winter barley (**Table 1**). Therefore, the region may be indeed related to both growth and drought tolerance. Four potential candidate genes were detected (**Supplementary Table S7**): *pmei3*, *HvDof9*, and *ABF1* encoding for a bZIP transcription factor (Sarkar and Lahiri, 2013). In rice, *OsABF1* was induced by abiotic stresses and connected with enhanced drought tolerance (Hossain et al., 2010). Additionally, a barley homolog to *WUSCHEL-RELATED HOMEOBOX5* (*WOX5*) is located in 2.5 cM distance, involved in stem cell maintenance in the root apical meristem (Sarkar et al., 2007). It was shown that enhanced levels of cytokinin downregulate key root tip genes, including *WOX5* and promote cell division in the root apical meristem (Zhang et al., 2013).

### Drought and Recovery-Adaptive Biomass QTL

In total, five biomass QTL are classified as drought or recovery-adaptive.

**Figure 5:**
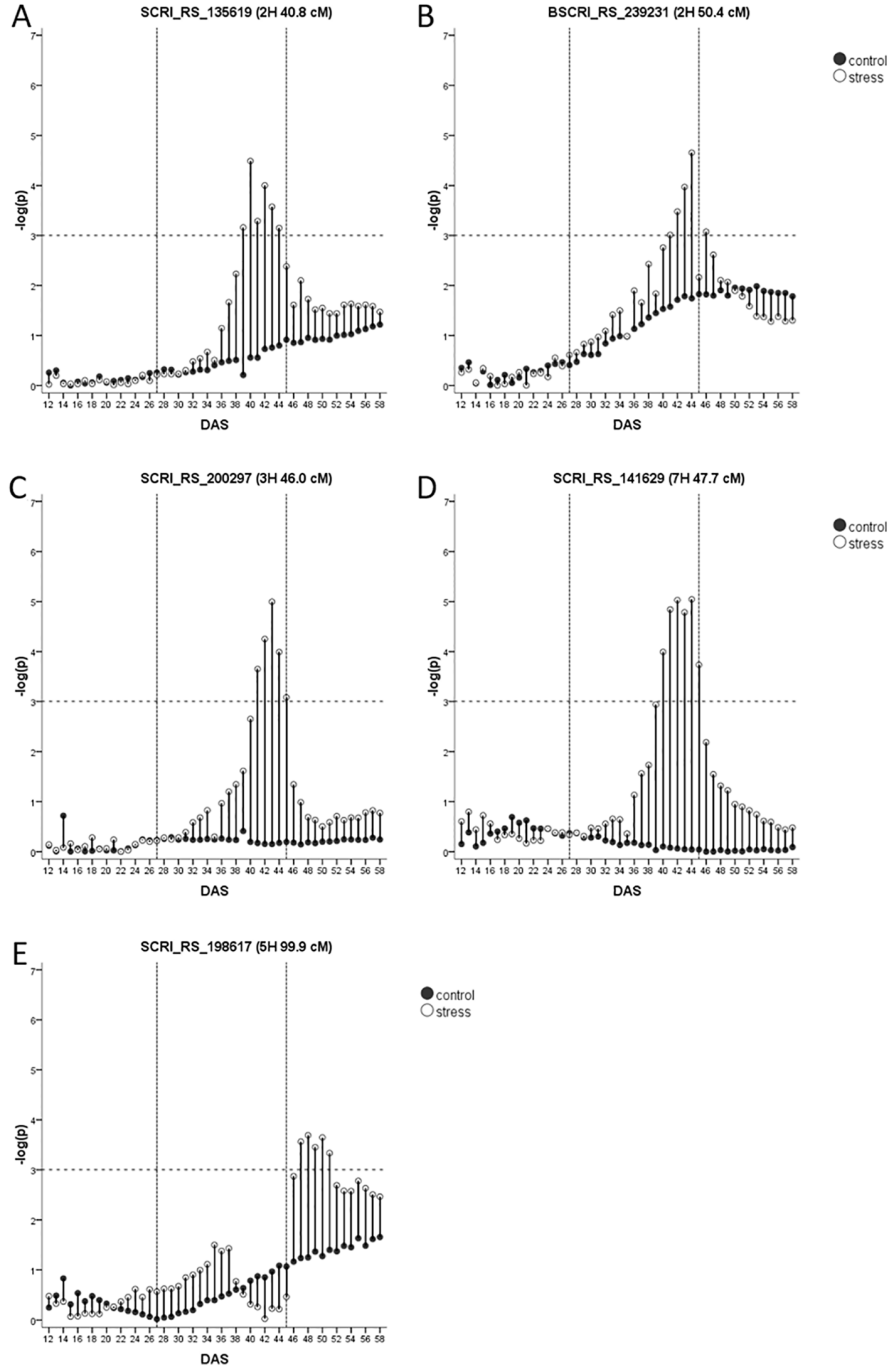
**(A**–**E)** Drought-adaptive biomass QTL with their –log(p)-values over time in days after sowing (DAS) in drought stress (unfilled) and well-watered (black) conditions. Presented is only the most significant SNP of each QTL (in case it consists of several SNPs). As the FDR is calculated for each day and in each treatment separately, the general significance level of –log(p)-value = 3 is indicated by a dashed horizontal line. The drought stress phase from DAS 27 to DAS 45 is indicated by the two dashed vertical lines.

Two drought-adaptive QTL on 2H (40.8 cM and 50.4 cM, **Figures 5A, B**), co-locate with QTL for biomass and agronomic traits (**Table 1**) and the genomic positions of the flowering time genes *HvCO18* and *HvFT4*, respectively. However, the two genomic regions harbor also genes related to drought tolerance (**Supplementary Table S7**). Within the vicinity of the QTL at 40.8 cM *HvHAK2*, a potassium transporter of the KUP6 family and a second potassium transporter that shows 65% sequence similarity with *HvHAK2* are located. For the QTL at 50.4 cM, a candidate could be MLOC_58500.1 with homology to the wheat heat transcription factor *TabZIP28* that is upregulated by heat and drought (Geng et al., 2016).

A very interesting drought-adaptive QTL in the centromeric region of 3H (**Figure 5C**) co-locates with the known flowering time gene *HvGI* (Dunford et al., 2005). The QTL further coincided with the only QTL identified for fAW at time A. In the full barley panel, several QTL for root system angle and root system depth were residing in this genomic region (Zhongtao et al., 2019). Moreover, in winter barley, a biomass QTL under WW conditions and a QTL for leaf color under drought stress were detected in this region (Wehner et al., 2015). *HvGI* is reported to have pleiotropic effects on other traits like plant height, grain yield, harvest index (Wang et al., 2010) and in terms of physical map position it is indeed the closest candidate gene (**Supplementary Table S7**). However, the centromeric region also harbors other potential candidate genes. At 44.1 cM the barley homolog of rice *CYP90D2/*D2is located. It encodes a cytochrome P450, that was reported to be completely deleted in the BR mutant *csdd1* (Li et al., 2013). Further, *HvCKX1* is located at 45.8 cM (Mameaux et al., 2012). In tobacco and *Arabidopsis*, downregulation of cytokinin through overexpression of *CKX1* led to increased drought tolerance (Lubovská et al., 2014; Prerostova et al., 2018). RNAi silencing of *HvCKX1* led to a higher yield caused by an increased number of spikes and seeds (Zalewski et al., 2014). Another very interesting drought-candidate is *HvABCG31/Eibi1* (46.3 cM) responsible for cuticle formation (Chen et al., 2011; Nevo, 2014). Variation in the promoter sequence of this gene was responsible for the variation in trait expression enabling wild barley plants to be more tolerant to drought conditions (Ma et al., 2012) and a drought-adaptive QTL in its vicinity encourages to study the effects of allelic variation within this genefor breeding purposes.

The last drought-adaptive QTL is located on 7HS at 47.7–48.3 cM (**Figure 5D**). It coincides with several QTL for seedling growth under control conditions and osmotic stress in the full barley panel and for leaf blade area (**Table 1**). QTL for biomass under stress and control conditions, and for DSI in winter barley (Wehner et al., 2015) are located in the same interval. A potential candidate could be a gene with 98% sequence similarity (**Supplementary Table S7**) to the type-B response regulator *ARR12a* of *Lolium perenne* (Roche et al., 2016). These transcription factors control cytokinin-dependent gene expression and are therefore critical for growth and the response to abiotic stress (Zubo et al., 2017). In close distance to the QTL, at 47.9 cM, resides an ethylene-responsive transcription factor (**Supplementary Table S7**).

The only recovery-adaptive QTL was detected on 5H at 99 cM (**Figure 5E**). It overlaps with the genomic region harboring the flowering time gene *HvPRR95* (**Supplementary Table S7**). Several agronomic QTL and QTL for seedling biomass and osmotic adjustment were mapped in its vicinity (**Table 1**). Another major gene in this region is *Vrs2*. One natural allele of *Vrs2* has been found correlated with plant development, leaf number, leaf area and tiller number in the full barley panel (Youssef et al., 2017) comprising two- and six-rowed genotypes. However, nothing is known about the effects of natural variation within one of the row type groups. Three more plausible drought-related candidates are located in the vicinity of the QTL. The first is *HvPap-1* (*HvSF42*) encoding for a cysteine protease (CP) active during flag leaf senescence (Díaz-Mendoza et al., 2014). Its expression was upregulated after two weeks of drought, and interestingly, *HvPap-1* knock-down lines showed an increase in cuticle thickness, stomata per area, ABA concentration and quantum efficiency of PSII under drought (Gomez-Sanchez et al., 2019). Accordingly, overexpression of the homologous wheat gene *TaCP* in *Arabidopsis* led to an increased survival after osmotic stress (Zang et al., 2010). The second drought-related candidate represents the heat stress transcription factor *HvHsfB2c*, playing a role in drought tolerance by possible control of expression of several subclasses of heat shock proteins (Reddy et al., 2014). A third drought-related candidate is the aquaporin *HvPIP2;7*.

### QTL for Drought Tolerance Related Traits Obtained From Biomass Growth Curves

The genetic architecture of traits obtained from growth curve models has been studied here for the first time.

**Figure 6:**
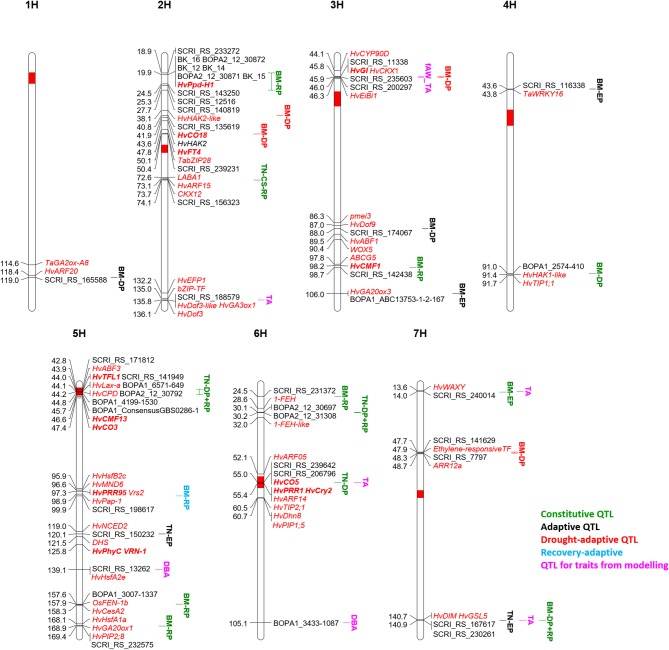
QTL map of the seven barley chromosomes 1H to 7H for classification of biomass (BM) and tiller number (TN) QTL along with QTL for traits from drought-growth pattern modelling: biomass at time A (DBA), time A (TA) and fraction of available water at time A (fAW_TA). The time period of significance of each QTL is indicated in the QTL name: early growth phase (EP), drought phase (DP), recovery phase (RP). All potential candidate genes are given in red and italic, the flowering time genes are additionally highlighted in bold.

The stop of growth (and the onset of wilting) is reflected by the modeled parameter time A, which proofed to be very sensitive in case growth conditions are not sufficiently controlled (Neumann et al., 2015). After adjustment of greenhouse conditions, time A showed a high heritability, and four QTL could be detected. The dependence of time A on initial biomass in this diverse collection resulted in two out of four QTL for time A co-locating with QTL for biomass (**Figure 6**). The advantage of early flowering and thereby low biomass as a drought escape mechanism in severe drought conditions is well known (Shakhatreh et al., 2001; Paul et al., 2016; Shavrukov et al., 2017). Nevertheless, two QTL for time A were not associated with biomass in our study. The first positioned on 2HL (135.8 cM) is the second most important QTL for time A in respect to the explained genetic variance. It coincided with a QTL for tiller number at DAS 58 in control conditions and for root traits and leaf blade area in the full barley panel (**Table 1**). Further, QTL for osmotic adjustment and for the DSI in winter barley (Wehner et al., 2015) earmark this region as interesting to search for candidate genes for growth and for the delay of wilting (**Supplementary Table S7**). In this context, we identified *HvGA3ox1* along with two copies of *HvDof3*, MLOC_73626.1 annotated as bZIP transcription factor, and *HvEFP1*, a member of the cysteine-rich EPIDERMAL PATTERNING FACTOR (EPF) family of secreted signaling peptides that is involved in stomatal development (Hughes et al., 2017). Barley plants overexpressing *HvEFP1* showed a slower water loss during a drought period in a comparable growth stage as in our study and had higher water use efficiency and seed yield (Hughes et al., 2017). A QTL for delayed onset of wilting in its vicinity should emphasize evaluation of barley germplasm collections for natural variation of *HvEFP1*.

The second QTL for time A is located on 6H at 55 cM and co-located with a QTL for tiller number at DAS 45 in both treatments, although coming from different SNPs. In the full barley panel, root QTL were located at the same position but also associated with different SNPs (Abdel-Ghani et al., 2019; Zhongtao et al., 2019). The centromeric region is reported to be associated with many QTL for agronomic traits and harbors several possible candidates, including three flowering time genes (**Table 1**). Further, *HvARF05* and *HvARF14* could be candidates for this locus. In winter barley, Wehner et al. (2015) observed with a QTL for osmotic adjustment under drought, which co-localizes with the QTL for time A. A candidate gene related to dehydration tolerance could be *HvDhn8* (**Supplementary Table S7**), mainly induced by drought stress (Tommasini et al., 2008). Dehydration tolerance could also refer to *HvTIP2;1* and *HvPIP1;5*.

For biomass at wilting time (DBA) no QTL passed the FDR. However, two out of six potential QTL (–log(p) values ≥ 3) were unique for this trait across both treatments: on 5HL at 139.1 cM and 6HL at 105.1 cM. Interestingly, a stress-specific QTL at the seedling stage was identified in the full barley panel at the same position of the QTL on 5HL, congruent with QTL for biomass and osmotic adjustment under drought stress in winter barley (**Table 1**). Two potential candidates were identified in a distance of 1 cM: the heat shock transcription factor *HvHsfA2e* and *Dhn9* (**Supplementary Table S7**). Also, the DBA-QTL on 6HL co-locates with QTL for biomass and DSI in winter barley (**Table 1**). Drought-related candidates in that area represent *Dhn4* and *Dhn7* (**Supplementary Table S7**).

Similar as for DBA, also for the re-growth rate *k* no SNP passed the FDR, although 15 SNPs had –log (p) values ≥ 3. Nevertheless, no unique locus was detected, and so, *k* seems not to yield additional information on re-growth on the genetic level.

### Genetic Architecture of Tiller Number Under the Influence of Drought Stress

In many cases, a higher number of tiller reflects higher biomass (Jaradat et al., 2005). Similar to biomass, the genetic architecture of tiller number changed over time according to earlier studies in rice (Yan et al., 1998; Abe et al., 2012; Alqudah et al., 2016) and wheat (Li et al., 2010). In total, we detected 14 QTL across both studies, six in the current study, and twelve in the control treatment (Neumann et al., 2017). Four QTL were found in the current study at DAS 27 in contrast to the previous study. The discrepancy may arise from the discussed differences in early plant development in both studies. As for biomass, we classified the tiller number loci into constitutive or adaptive QTL. In this way, four QTL were classified as constitutive (**Figure 6**), while ten QTL were classified as adaptive.

### Constitutive Tiller Number QTL

The first constitutive QTL for tiller number on 2HL at 74.1 cM was detected as a hotspot for root and shoot traits at seedling stage in the full barley panel and co-located with other agronomic QTL in different studies (**Table 1**). A candidate might be MLOC_52145.1 (**Supplementary Table S7**) with high similarity to maize *CKX12*, which was shown to be highly expressed in the maize shoot at the five-leaf stage (Gu et al., 2010). In rice, down-regulation of a *CKX* lead to an increased tiller number in transgenics in the field (Yeh et al., 2015). Another candidate gene (MLOC_6463.1) has high similarity to *LABA1* in rice (**Supplementary Table S7**). *LABA1* is involved in the final step of bioactive CK biosynthesis. In rice, *LABA1* is responsible for awn frequency and awn length mediated by CK in the awn primordia (Hua et al., 2015). A function of this gene for tillering in barley remains speculative, but also expression of *MONOCULM 3* in rice was induced by cytokinin, a gene known to be required for axillary bud formation (Lu et al., 2015). Moreover, *HvARF15* is located at 73.1 cM. In rice, miR167 is regulating ARFs, and miR167 overexpressing lines showed a lower tiller number in the vegetative stage in connection with downregulation of four *OsARFs* (Liu et al., 2012).

Tiller number at DAS 45 and 58 in the control treatment, and tiller number at all three time points under drought stress were mapped to the centromeric region of 5H, which harbors the gene *HvLax-a* (Jost et al., 2016). Together with its paralogue of *Hvre-t4* (*Uniculm 4*) (Tavakol et al., 2015) is known to control tillering in barley. Accordingly, QTL for tiller number were also mapped to the same region in the full barley panel (**Table 1**) and in a different barley collection (Mora et al., 2016). Many further agronomically important genes and QTL map to the centromeric region (**Table 1**).

The most important locus for tiller number in both treatments is located on 6HS at 30.1-30.2 cM. It explained a high proportion of the genetic variance in both studies and was significant at DAS 45 and 58, while for DAS 27 it was only found significant in the current study. In close proximity, we also detected a constitutive biomass QTL at 24.5 cM. *1-FEH*, a potential candidate for biomass and tiller number is located at 28.6 cM, between these two QTL (**Supplementary Table S7**).

The fourth constitutive QTL for tiller number on 6H at 55 cM was significant in both studies only at DAS 45. Accordingly, this QTL was earlier identified by Alqudah et al. (2016) within the two-rowed germplasm at the stage of awn primordium and tipping but not later. From a breeding perspective, stage-specific QTL might provide opportunities for genetic manipulation to maximize the number of tillers. The genomic region harbors flowering (*HvCO5, HvPRR1, HvCry2*) and growth-related genes (*HvARF14*, **Supplementary Table S7**).

### Adaptive QTL for Tiller Number

Contrary to biomass, not a single adaptive QTL for tiller number was detected under drought stress. Two QTL were identified at DAS 27 in the current study, but being present only at the start of stress they can’t be regarded as drought-adaptive. The first of these two (5H 120.1 cM) was previously identified by Alqudah et al. (2016) for tiller number at the time of tipping. Within 5 cM distance *Vrn-H1* and *HvPhyc* are located and may have pleiotropic effects on tiller number (**Supplementary Table S7**). However, the QTL region contains also *HvNCED2* (119.0 cM), a gene involved in ABA biosynthesis (Wang et al., 2017) and ABA is involved in bud outgrowth (Kebrom et al., 2013). Further, MLOC_51599.3 with high sequence similarity to the wheat deoxyhypusine synthase (DHS) is located at 121.5 cM. DHS is involved in the activation of the eukaryotic translation initiation factor eIF5A that is essential for cell division (Tiburcio et al., 2014).

The second QTL for tiller number at DAS 27 is identical to the constitutive biomass QTL on 7HL at 140.9 cM. In the six-rowed subpanel, a QTL for non-productive tiller number at maturity was detected here (Alqudah et al., 2016). The current study shows that this QTL is also linked to tiller number in two-rowed spring barley at early developmental stages.

The eight remaining adaptive QTL for tiller number were identified only in the control treatment at DAS 45 and/or 58. They are located on 2H (58 cM, 124.9 cM, 135.8 cM), 5H (30.6 cM), 6H (24.5 cM), 7H (68 cM, 120.4 cM, 134.2 cM) (Neumann et al., 2017).

## Summary

Non-invasive phenotyping allows for resolving the timing of QTL appearance. It can resolve which genetic loci are responsible for early growth vigor, growth *per se*, drought tolerance, and recovery from stress. The genetic architecture of biomass during the drought period was altered strongly compared to well-watered conditions. Drought tolerance related loci appeared mainly after the arrest of growth, reflected by the parameter time A obtained from growth modeling. During plant recovery, mainly a switch back to the genetic architecture observed under well-watered conditions was seen. After the end of drought, the *HvPPD-H1*-region revealed the highest effect, unlike in the well-watered treatment, where the *HvDIM*-locus was most prominent during the same time. In general, co-localisation of biomass and flowering time genes was expected and confirmed by the detection of candidate genes including *HvCO18, HvFT4, HvGI, HvCMF1*, or *HvPRR95*. However, also genes involved in dehydration tolerance were often located in QTL regions. No drought-related QTL were detected for tiller number, where the change in genetic architecture was marked by the disappearance of QTL compared to well-watered conditions. Our comprehensive case-study in barley demonstrates the potential of non-invasive phenotyping for resolving the genetic architecture of complex traits that changes throughout development and under contrasting growth conditions. Traits are highly heritable even if drought stress is applied — as long as conditions are controlled and strictly standardized. In future studies, replicates within experiments can be even further decreased, allowing for an increase in the number of genotypes and a concomitant increase in the power of genome-wide association scans. The drought and growth-related genes identified as candidates in the present study represent informed targets for further validation by re-sequencing in our barley panel. Natural variation is already known for *HvABCG31/Eibi1, HvDof3*, and *HvPap-1*. Drought-related QTL in the vicinity of these genes highlight their potential value as starting points to breed for enhanced drought tolerance.

## Data Availability Statement

The datasets generated for this study can be found in the e!DAL – Plant Genomics & Phenomics Research Data Repository, DOI: 10.5447/IPK/2019/14. All other data are available on request to the corresponding author if not included in the **Supplementary Files**.

## Author Contributions

SD and KN designed the study, conducted the experiments, and analyzed phenotypic data. DC modeled the biomass growth curves, MG evaluated fAW. GL and YZ analyzed performed statistical analysis and GWAS. SD and KN were the major contributors in writing the manuscript. JR, BK, and AG were involved designing the study, data interpretation and writing of the manuscript. All authors read and approved the final manuscript.

## Funding

This work was conducted as part of the CROP.SENSe.net (FKZ 0315530E) and BARSELECT (FKZ 0315969D) consortia, both funded by the Federal Ministry of Education and Research of Germany (BMBF). We also thank the Humboldt foundation for support of Michele Grieco.

## Conflict of Interest

The authors declare that the research was conducted in the absence of any commercial or financial relationships that could be construed as a potential conflict of interest.

## Abbreviations

DAS, days after sowing.
