# Supplementary material for: Non-Invasive Phenotyping Reveals Genomic Regions Involved in Pre-Anthesis Drought Tolerance and Recovery in Spring Barley
Source: Front Plant Sci. 2019 Oct 25;10:1307. doi: 10.3389/fpls.2019.01307 (PMC6823269; doi:10.3389/fpls.2019.01307)
Supplement: Supplementary file 1 [file DataSheet_1.pdf]

Supplementary Material

Non-invasive phenotyping reveals genomic regions involved in pre-anthesis drought tolerance and recovery in spring barley

Sidram Dhanagond[1], Guozheng Liu[1,2], Yusheng Zhao[1], Dijun Chen[3], Michele Grieco[1], Jochen Reif[1,5], Benjamin Kilian[1,4], Andreas Graner[1,5], Kerstin Neumann[1*]

[1]Leibniz Institute of Plant Genetics and Crop Plant Research (IPK), Seeland, Germany

[2] European Wheat Breeding Center, BASF Agricultural Solutions Seed GmbH, Seeland, Germany

[3] Institute for Biology, Humboldt-Universität zu Berlin, Berlin, Germany

[4] Current address: Global Crop Diversity Trust (GCDT), Bonn, Germany

[5] Martin-Luther-University Halle-Wittenberg, Halle/Saale, Germany

***Correspondence: Kerstin Neumann**

E-mail: neumannk@ipk-gatersleben.de

Determination of fraction of plant available water (fAW)

Field capacity (FC) is the water content of water-saturated soil, after percolation. It depends on the soil type used and has to be determined accordingly for each soil.

FC was determined by the difference between the weight of water-saturated soil and oven-dried soil for the substrate that is used in the current study (*Neumann et al.* 2015). However, in practice, plants are unable to extract water down to 0% water content in the soil. To determine the fraction of (plant) available water (fAW) in our soil type, a calibration experiment was conducted in early 2017 on the LemnaTec-Scanalyzer 3D system, including 38 spring barley lines covering the full range of biomass from previous study (Neumann et. al. 2017). The experimental drought set-up comprised 3 plants per genotype, resulting in a total of 114 plants. Stress was applied at 27 days after sowing (DAS) by complete water withdrawal until experiment end. Potting, temperature, and light settings in the greenhouse were the same as in the current study. Plants were observed until their recorded weights reached a plateau (until DAS 63), thus implying a drought period of 36 days. Plant fresh (FW) and dry weight (DW) were measured at DAS 64. From the recorded total weights of each carrier (Fig. S1A, B), the soil weight value corresponding to fAW 0% was obtained for each plant: from the recorded total carrier weight during the imaging run of DAS 63, all equipment (pot, carrier, plant support, blue sand) and the individual plant FW was subtracted. The average of the obtained soil weight at fAW 0% was 353.8 g and thereby 53.8 g higher compared to the weight of oven dried soil (300g). We tested if the experimentally determined soil weight at fAW 0% was affected by genotype and performed an ANOVA for this trait. The effect of genotype was not significant, indicating that there are no genotypic differences down to which point barley genotypes can extract water. The estimated average soil value at experimentally determined fAW 0% was used to calculate the fraction of plant available water (fAW) in the soil amount used in our experiments. Based on that, we also calculated the percentage of fAW at our watering target weights for both treatments.

Image capturing

Images were captured in 4 different angles – one top view (from DAS 1) to DAS 58), three side views *viz.* at 0°, 45° & 90° angle from DAS 12 to DAS 58 (when plant size were appropriate for side view imaging). The resolution of the digital camera (Basler AG, Germany) capturing images in visible light range was 1628 × 1236 pixels, with a pixel size of 4.4 × 4.4 μm. Images were analyzed by Intermittent technical issues caused unexpected interruptions of the daily run and led to incomplete or no data. Hence, in experiment 1, the data was incomplete or missing for DAS 14 and 35, in experiment 2 for DAS 24, and in experiment 3 on DAS 52 and 53. The image processing pipeline, the IAP software, is available at http://iapg2p.sourceforge.net/.

Modelling of biomass growth patterns

A bell-shaped model ($y = Ae^{bt-at^2}$) was found as good fit for biomass curve until the re-watering and two parameters were extracted: wilting time 'TA' ($TA = \frac{b}{2a}$) and digital biomass at TA (DBA). For biomass development in the recovery phase from DAS 46 to 58 a simple linear model ($y = a + bt$) was fitted and the re-growth rate (k) was determined to quantify the speed of recovery.

Determination of plant available water fraction at Time A

For every plant, fAW at time A (TA) was estimated by linear regression using the average value of maximum fAW from the calibration experiment. In the curve describing the time-course of fAW along DAS, linear regression was carried out in the range of the corresponding TA ± 1 DAS obtained from the bell-shaped wilting model. By interpolation at the corresponding TA, fAW at TA was obtained. This procedure allowed also to compensate for the technical error associated to fAW estimation.

Seasonal differences between experiments in relation to tipping time stage (BBCH 49)

Tipping time (BBCH 49) in greenhouse experiments is influenced by seasonal differences. In both treatments majority of plants reached tipping stage in the experiment closest to the field growing period (experiment 1 in WW and experiment 2 in DS). In WW, tipping time could be assessed for most plants in experiments 1 and 2 but not in 3 (ending in November). Under DS majority of plants didn't reach tipping in experiments 1 and 3 (ending in September). In an

attempt to estimate the influence of phenology on traits obtained under DS we used tipping time BLUEs from Neumann et al. (2017) for correlations (Fig. S14 and S15).

Table S1 Schedule of experiments for biomass formation under drought stress with start and end dates of phenotyping.

Experiment	Date of sowing	Date of last imaging
Experiment 1	05.03.2013	02.05.2013
Experiment 2	14.05.2013	11.07.2013
Experiment 3	23.07.2013	19.09.2013

Table S2 Descriptive statistics - Minimum (Min), maximum (Max) mean, standard deviation (SD) and coefficient of variation (CV) were estimated with BLUEs of single experiments. DBA – digital biomass at time A in 10^6 Voxel, TA – time A in days after sowing (DAS), fAW - fraction of plant available water at TA, *k* - re-growth rate in 10^6Voxel/day, FW – fresh weight in g, TN – tiller number at DAS 27, 45 and 58.

Exp	Trait	Min	Max	Mean	SD
**1**	**DBA**	8.49	17.77	12.78	1.65
**2**	**DBA**	8.74	19.76	15.84	1.65
**3**	**DBA**	13.73	24.13	19.29	1.81
**1**	**TA**	34.30	41.90	36.57	1.19
**2**	**TA**	34.60	41.90	36.34	0.99
**3**	**TA**	35.40	44.30	37.96	1.43
**1**	**fAW_TA**	12.67	27.23	21.25	2.75
**2**	**fAW_TA**	14.78	28.24	21.85	2.80
**3**	**fAW_TA**	13.60	26.55	20.66	2.29
**1**	***k***	0.003	0.058	0.033	0.010
**2**	***k***	0.001	0.045	0.024	0.009
**3**	***k***	0.004	0.051	0.033	0.009
**1**	**FW59**	33.00	114.00	87.98	12.49
**2**	**FW59**	28.00	95.00	73.34	13.07
**3**	**FW59**	36.00	102.00	80.94	11.97
**1**	**TN27**	4.20	13.20	8.32	1.71
**2**	**TN27**	3.20	11.0	7.13	1.14
**3**	**TN27**	3.60	8.60	6.08	0.88
**1**	**TN45**	5.60	23.20	11.50	2.54
**2**	**TN45**	4.20	17.80	9.16	2.01
**3**	**TN45**	4.60	16.20	9.22	1.67
**1**	**TN58**	11.60	49.25	24.00	5.55
**2**	**TN58**	8.80	41.60	24.51	4.90
**3**	**TN58**	11.20	47.20	22.26	5.04

Table S3 Descriptive statistics Minimum (Min), maximum (Max), mean, standard deviation (SD) and coefficient of variation (CV) were estimated with BLUEs across all 3 experiments. DB – digital biomassin 10^6 Voxel, FW – fresh weight in g, TN – tiller number, TA – time A in days after sowing, DBA – digital biomass at time A in 10^6 Voxel, fAW - fraction of plant available water, k- re-growth rate in 10^6 Voxel/day.

Trait	Min	Max	Mean	SD	CV
**DB12**	0.18	0.48	0.34	0.07	0.20
**DB27**	3.70	89.33	69.39	9.83	0.14
**DB46**	11.62	25.88	21.15	2.18	0.10
**DB58**	15.83	79.24	55.01	11.39	0.21
**FW59**	32.32	99.72	80.75	11.64	0.14
**TA**	35.42	41.72	36.96	1.02	0.03
**DBA**	10.42	19.54	15.98	1.42	0.09
**fAW_TA**	15.15	25.89	21.23	2.02	0.09
k	0.003	0.051	0.030	0.009	0.29
**TN27**	3.67	10.47	7.18	1.02	0.14
**TN45**	4.80	19.07	9.96	1.90	0.19
**TN58**	10.53	46.02	22.59	4.94	0.22

Table S4 Correlations based on BLUEs across experiments among DB at the start (DAS27) and one day after re-watering (DAS46) and at the end of recovery period (DAS58), TA – time A, DBA – digital biomass at time DBA, fAW-TA – fraction of extractable soil water at TA, k - re-growth rate and DB at the start (DAS27) and one day after re-watering (DAS46) and at the end of recovery period (DAS58), TN – tiller number. Only significant correlations (p<0.05) are presented.

	**DB27**	**DB46**	**DB12**	**TA**	**DBA**	**fAW_TA**	**k**	**TN27**	**TN45**
**DB27**									
**DB46**	0.55								
**DB12**	0.74	0.27							
**TA**	-0.80	-0.31	-0.55						
**DBA**	0.75	0.84	0.41	-0.40					
**fAW_TA**	n.s.	n.s.	n.s.	-0.52	n.s.				
**k**	0.48	0.67	0.23	-0.42	0.62	0.27			
**TN27**	0.67	0.47	0.35	-0.62	0.59		0.49		
**TN45**	0.45	0.32	n.s.	-0.44	0.47	0.28	0.57	0.82	
**TN58**	0.31	0.23	n.s.	-0.31	0.40	n.s.	0.44	0.70	0.92

Table S5 is provided as separate excel-file. It contains all markers that were passing the FDR level for minimum one trait along with their position in the genome and all –log(p)-values for all further traits. All –log(p)-values>3 are highlighted in yellow, while numbers given in red text refer that the FDR was passed.

Table S6 Schedule of experiments for biomass formation in the well-watered experiments of Neumann et al. (2017) with start and end dates of phenotyping.

**Experiment**	**Date of sowing**	**Date of last imaging**
Experiment 1	08.05.2012	05.07.2012
Experiment 2	20.07.2012	16.09.2012
Experiment 3	25.09.2012	25.11.2012

Table S7 is provided as separate excel-file. Important QTL regions were screened for candidate genes using BARLEX (Colmsee et al. 2015) and blasted in NCBI. All genes that could be a likely candidate are listed with the genetic and physical distance to the representative SNP of the corresponding QTL.

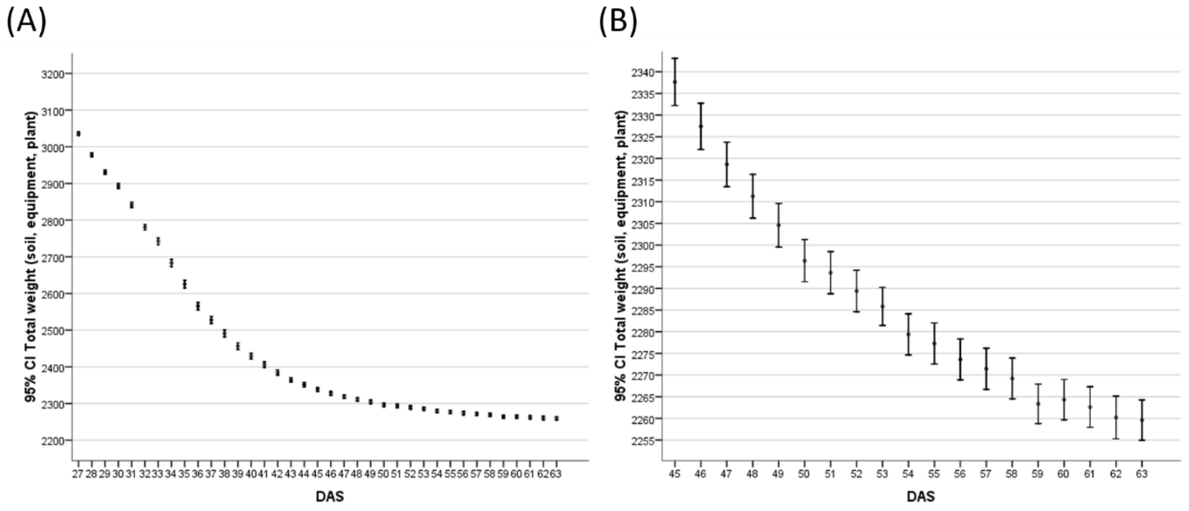

Fig. S1 Determination of the fraction of plant available water (fAW) **A)** Decrease of total weights during the drought period in days after sowing (DAS) and **B)** for better visualization in more detail for late drought period DAS 45 – 63

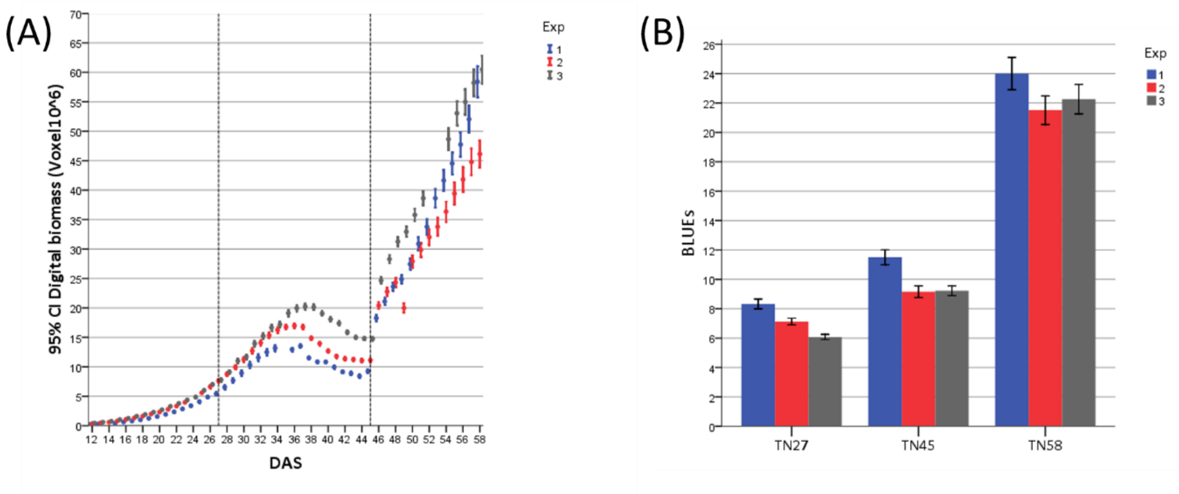

Fig. S2 BLUEs of single experiments (Exp 1 in blue, Exp 2 in red and Exp 3 in grey) in three experiments under vegetative-drought stress treatment **A)** Accumulation of image-based estimated 'digital biomass' (in 10^6Voxel) is shown from 12 to 58 days after sowing (DAS). Error bars represent the 95% confidence interval (CI). The drought stress period between DAS 27 and 45 is indicated by vertical dashed lines. **B)** Tiller number (TN) at three time points with error bars (95% CI): TN27 (beginning of drought period), TN45 (end of drought period), TN58 (end of recovery period).

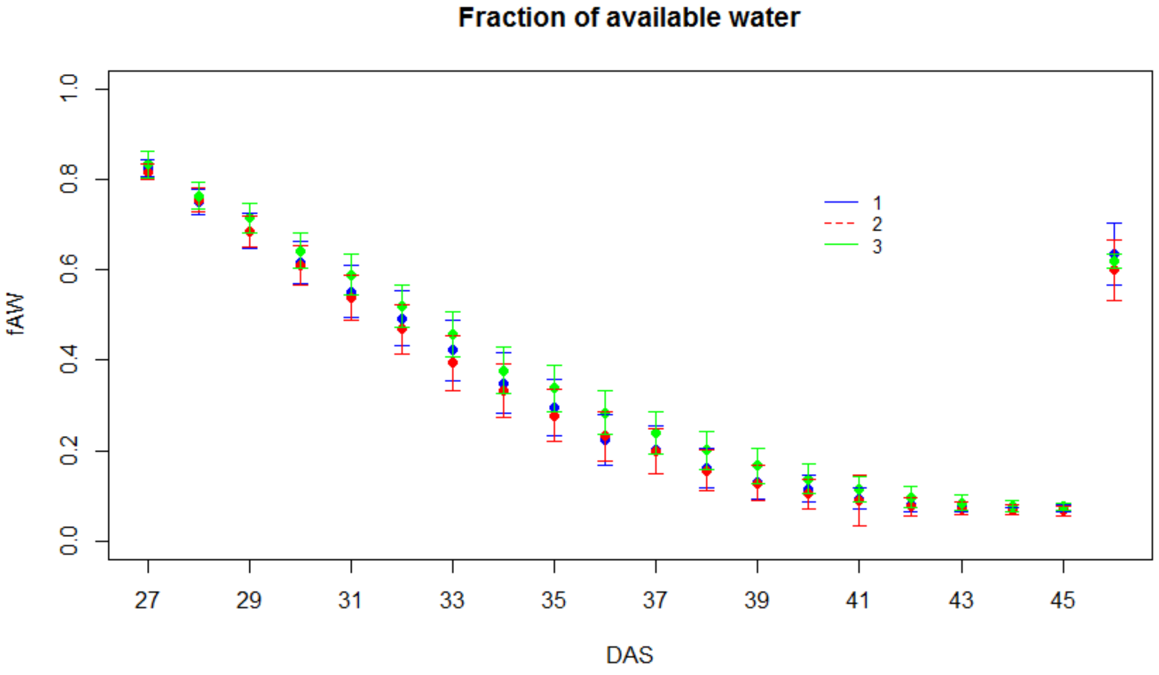

Fig. S3 Loss of water calculated as fraction of (plant) available water (scaled from 0 to 1) within the three experiments over the time of the drought stress phase until one day after re-watering. DAS = days after sowing. Calculations are done with the weight *before* watering, therefore fAW at re-watering day (DAS 45) is still in the drought phase.

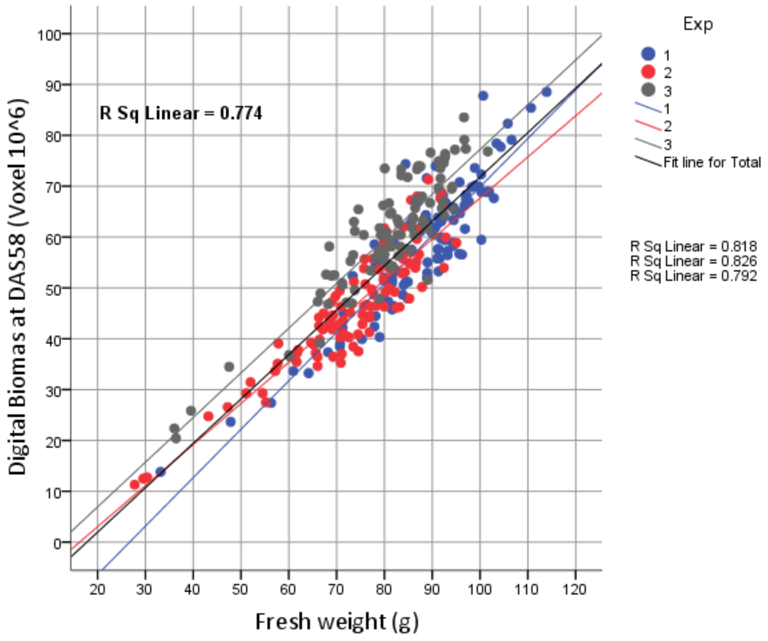

Fig. S4 Relationship of estimated digital biomass at the last day of imaging (DAS 58) against fresh weight (FW) of shoot biomass (DAS 59) among 99 genotypes within stress treatment, based on BLUEs of single experiments (Exp 1 in blue, Exp 2 in red and Exp 3 in grey) and their respective coefficients of determination R^2 on the right side. The R^2 across all BLUEs is shown in black color in the upper left side of the graph.

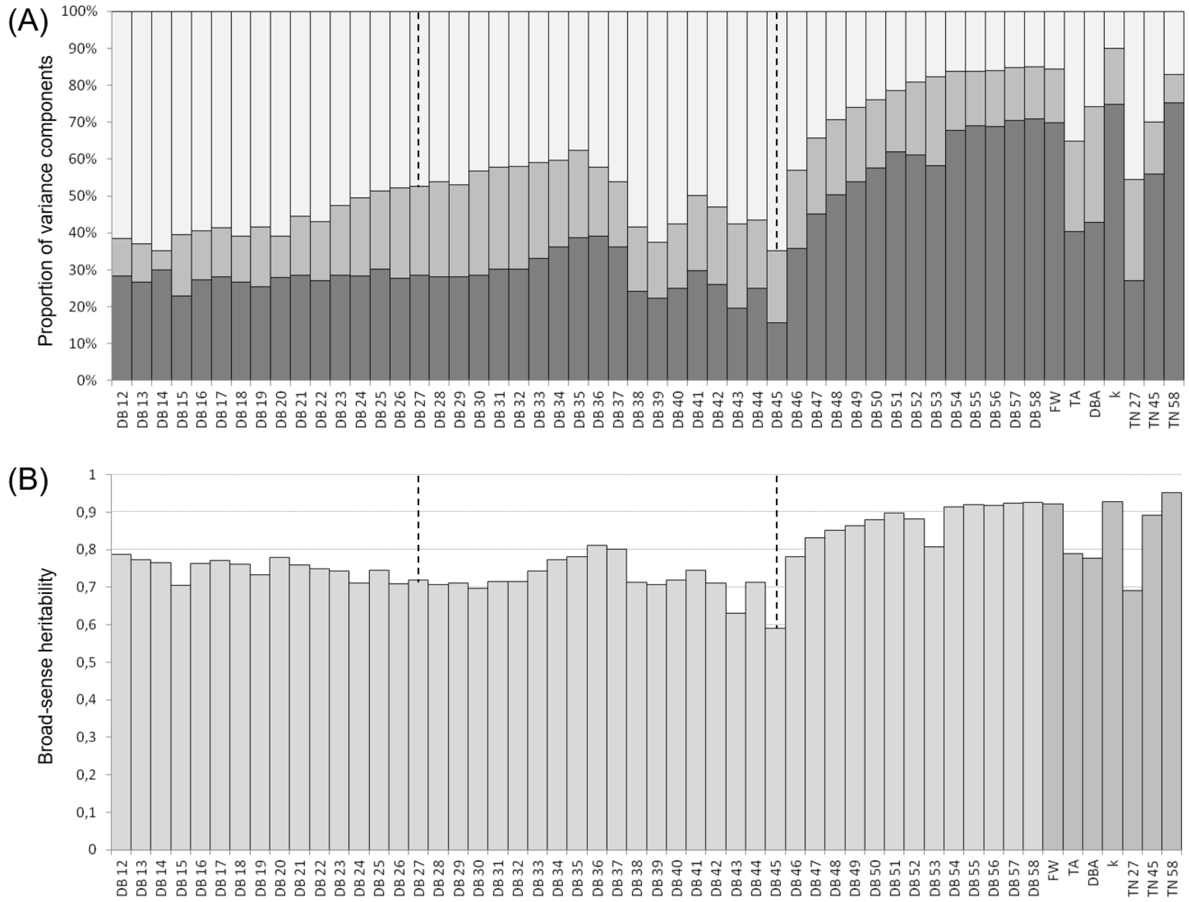

Fig. S5 A) Proportion of variance components for each time point of DB (DB12 to DB58), FW, TA, DBA, k, TN at three time points (TN 27, TN 45 and TN 58). The vertical dashed black lines represent the stress phase (27 DAS to 45 DAS). Dark grey – Genotypic variance, grey – Genotype x Environment interaction, light grey – unexplained variance. **B)** Broad sense heritability values (H^2) for DB (DB12 to DB58) in light grey, and in darker grey for FW, TA, DBA, k, TN 27, TN 45 and TN 58. The vertical dashed black lines represent the stress phase (DAS 27 to DAS 45).

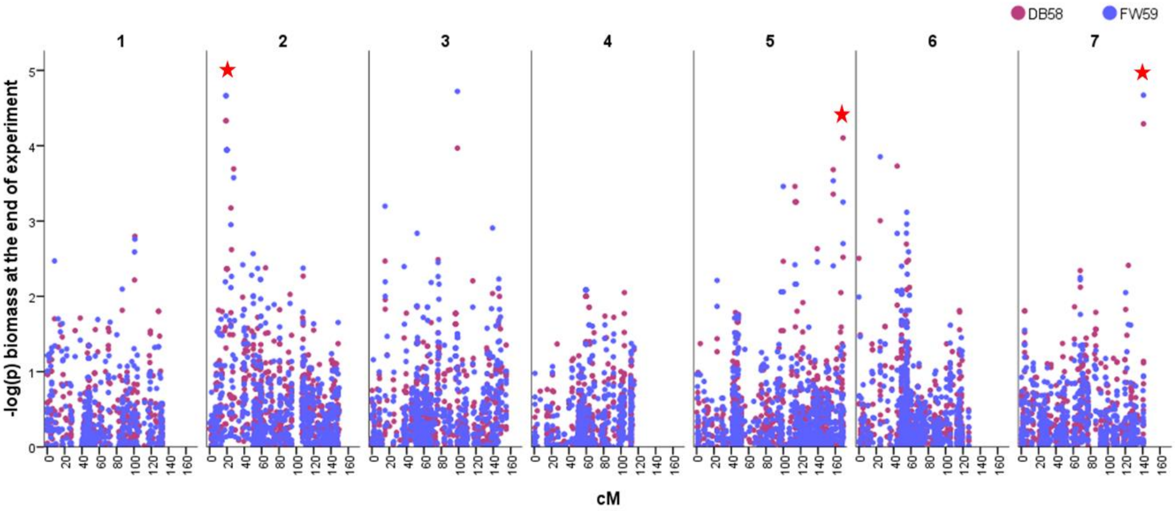

Fig. S6 Manhattan plot of genome-wide association mapping results for digital estimated biomass (DB) at the last day of imaging (DAS 58) and the destructively measured fresh weight (FW) one day later (DAS 59). The loci passing the false discovery rate (<0.1) for DB are indicated by a red asterisk. Numbers from 1 to 7 at the top refer to the seven barley chromosomes.

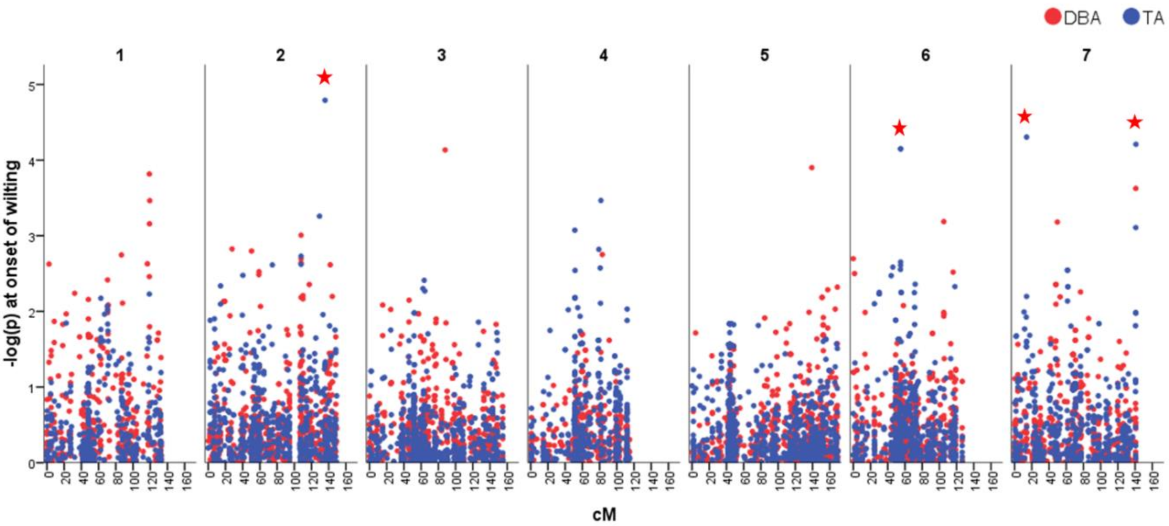

Fig. S7 Manhattan plot of genome-wide association mapping results for time A (TA) and biomass at TA (DBA) during the drought stress phase. The loci passing the false discovery rate (<0.1) are indicated by a red asterisk. Note, no loci for DBA passing the FDR were detected. Numbers from 1 to 7 at the top refer to the seven barley chromosomes.

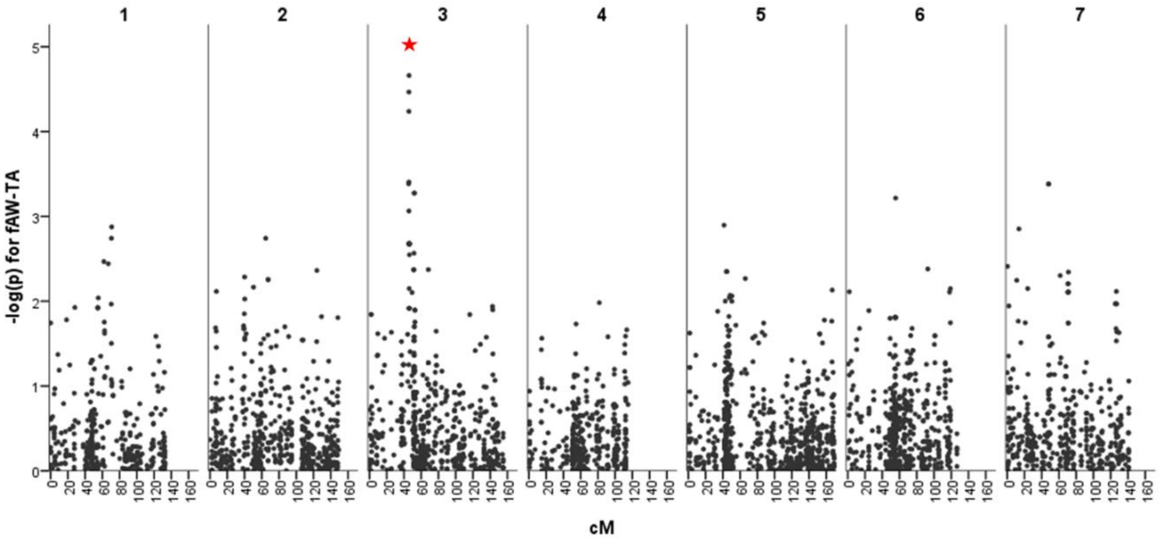

Fig. S8 Manhattan plot of genome-wide association mapping results for fAW_TA during the drought stress phase. The loci passing the false discovery rate (<0.1) are indicated by a red asterisk. Numbers from 1 to 7 at the top refer to the seven barley chromosomes.

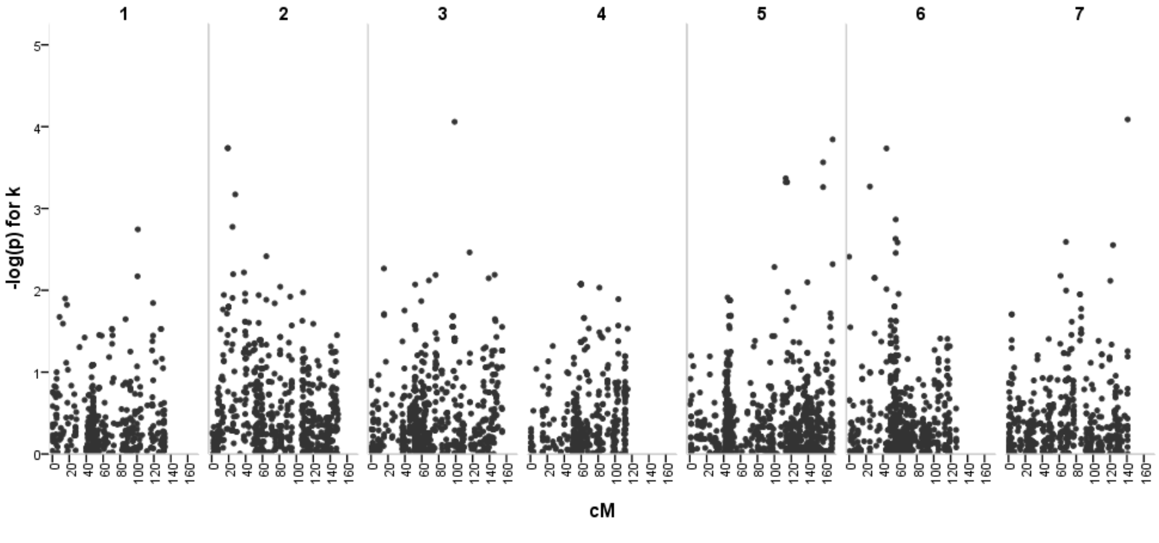

Fig. S9 Manhattan plot of genome-wide association mapping results for the re-growth rate *k* during the recovery phase. Note, no loci for k passing the FDR were detected. Numbers from 1 to 7 at the top refer to the seven barley chromosomes.

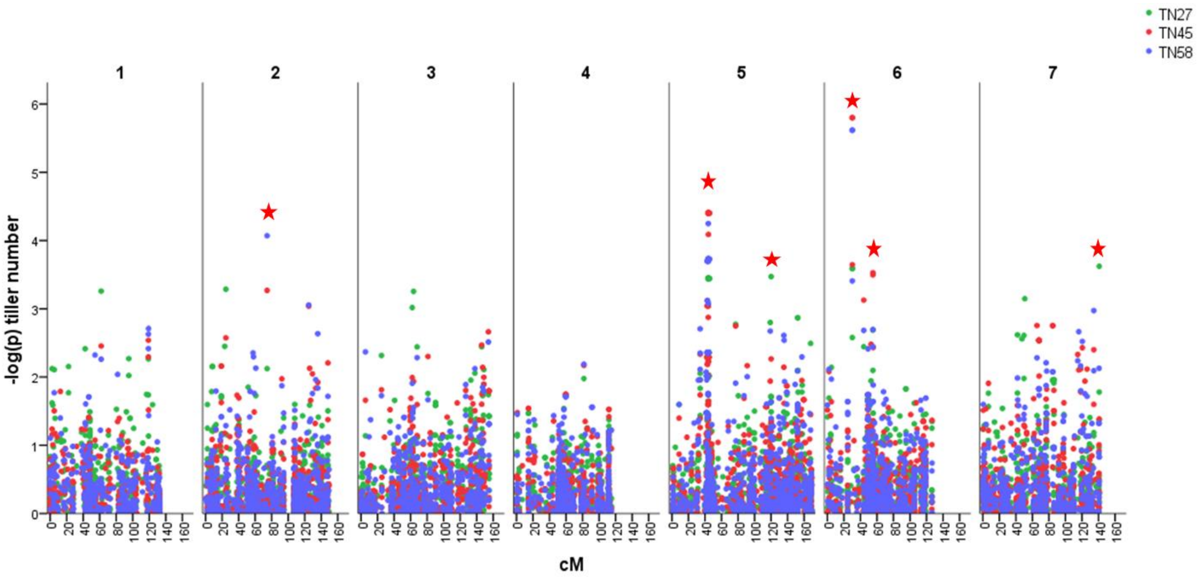

Fig. S10 Manhattan plot of genome-wide association mapping results for tiller number (TN) at DAS 27, 45 and 58. The loci passing the false discovery rate (<0.1) are indicated by a red asterisk. Numbers from 1 to 7 at the top refer to the seven barley chromosomes 1H to 7H.

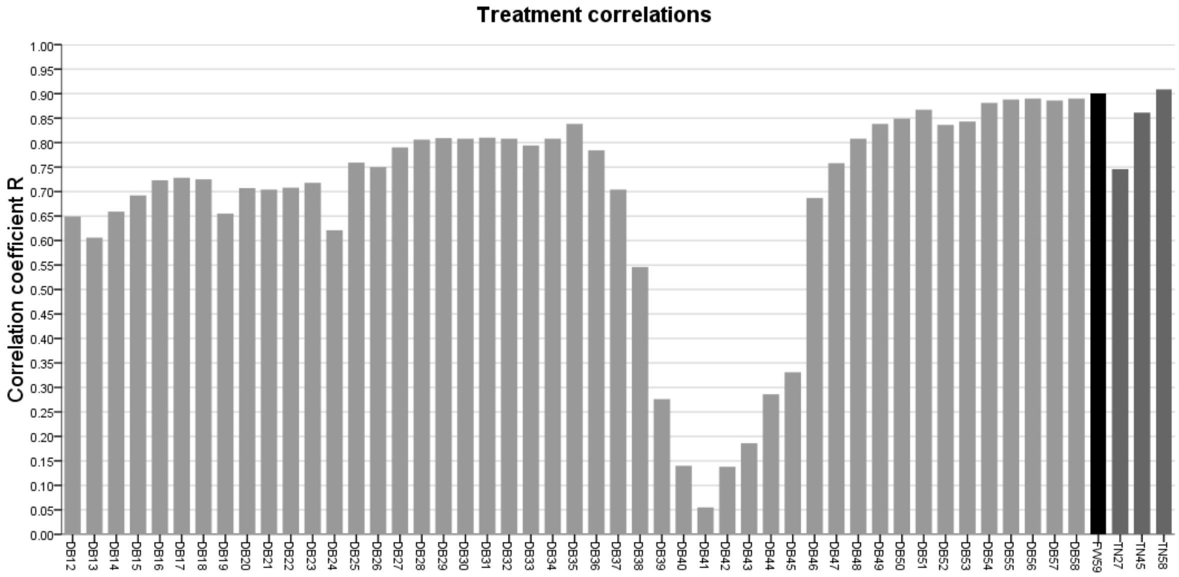

Fig. S11 Phenotypic correlation based on BLUEs across experiments between well-watered conditions (Neumann et al. 2017) and drought stress conditions. DB – digital biomass (light grey) the number behind indicates the day in terms of days after sowing (DAS), FW – fresh weight (black) at DAS 59, TN – tiller number (dark grey) at DAS 27, 45 and 58.

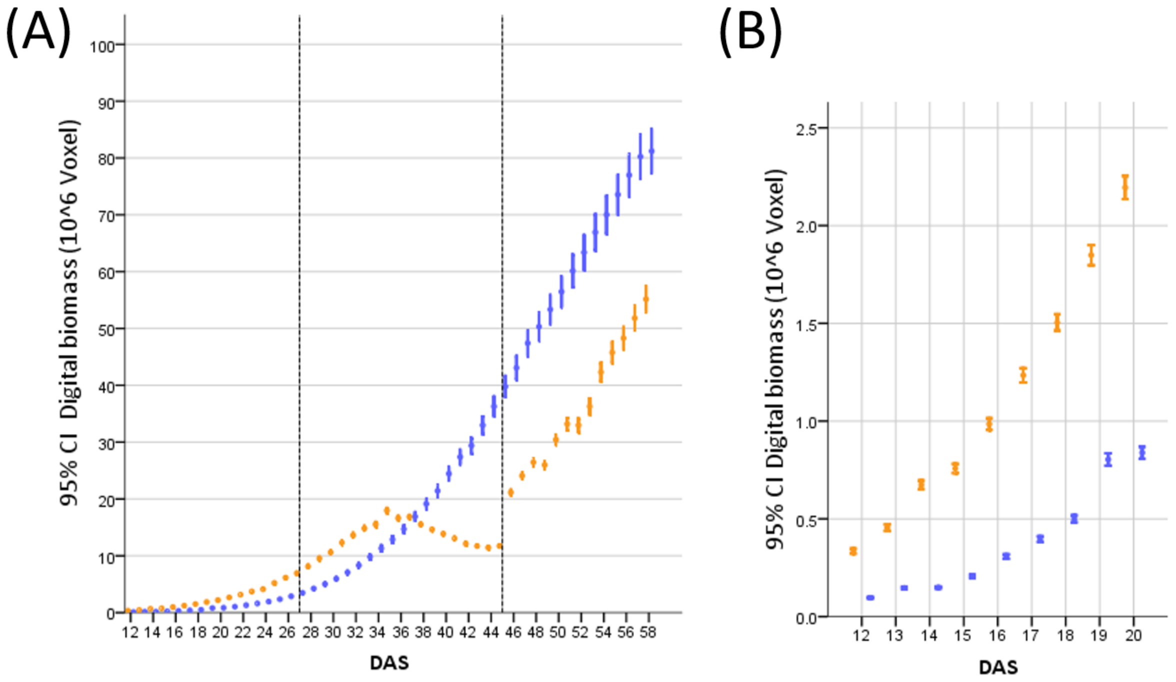

Fig. S12 Biomass growth in well-watered (blue) and in drought stress (orange) conditions based on BLUEs across experiments at the first common day of biomass assessment in both studies (DAS 12) **A)** 12 to 58 days after sowing (DAS). Error bars represent the 95% confidence interval (CI). The drought stress period between DAS 27 and 45 is indicated by vertical dashed lines and **B)** for better visualization in more detail for early biomass from DAS 12 – 20.

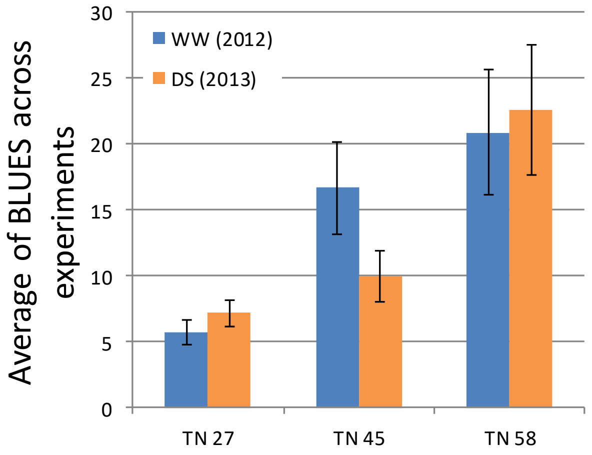

Fig. S13 Tiller number (TN) at DAS 27, 45 and 58 in well-watered treatment (blue) of Neumann et al. (2017) and in drought stress (orange) conditions based on BLUEs across experiments. Error bars represent the 95% confidence interval.

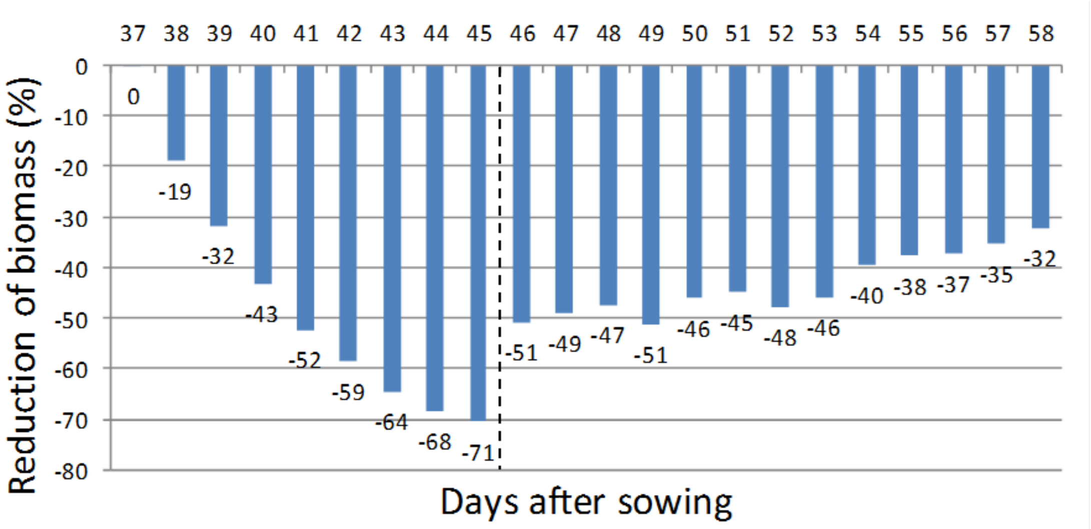

Fig. S14 Biomass reduction in the drought stress experiments compared to the complementary experiments under well-watered conditions (Neumann et al. 2017) based on the average of BLUEs across experiments in each treatment from DAS 37 (= average time A) to the end of the recovery period (DAS 58). The dashed vertical line indicates the end of the drought stress period.

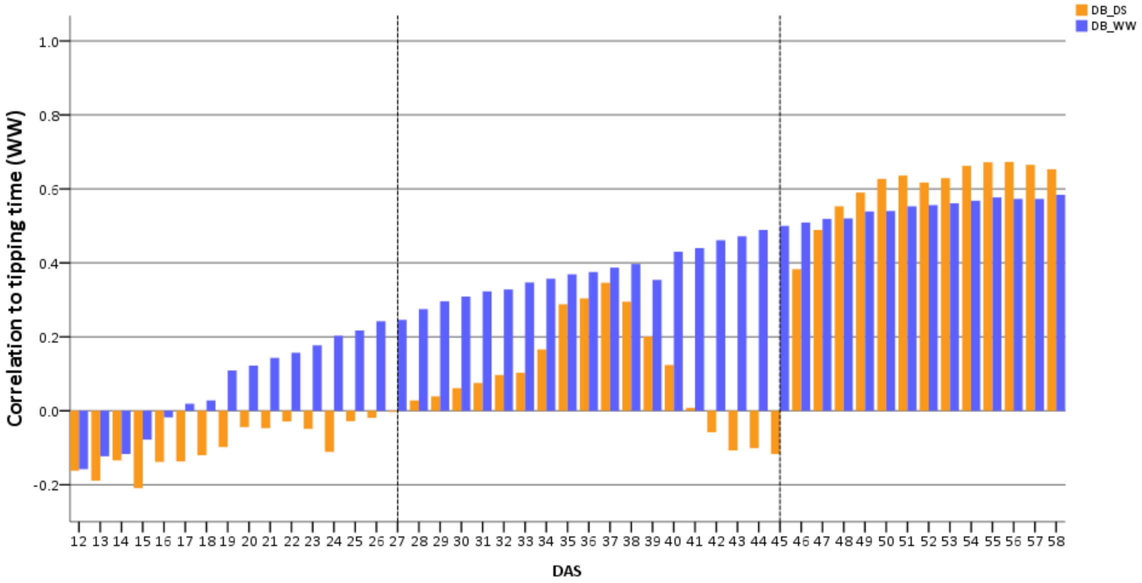

Fig. S15 Correlation of digital biomass (DB) in drought stress (DS) and in well-watered treatment (WW) to the tipping time (BBCH 49) from well-watered treatment (WW) of Neumann et al. (2017), all based on BLUEs across the respective experiments.

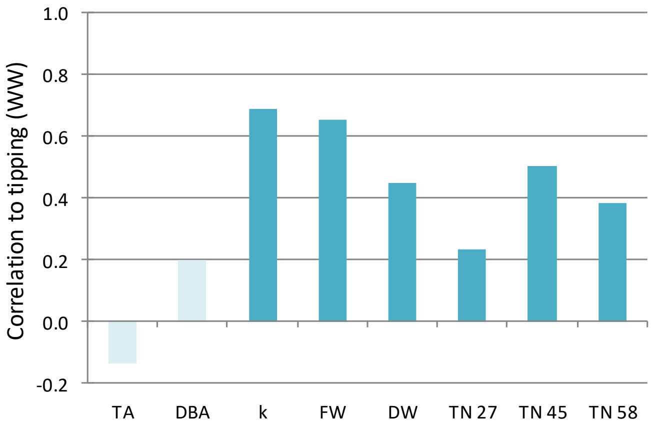

Fig. S16 Pearson correlation coefficient R of manual and modelled traits in the drought stress treatment to tipping time (BBCH 49) from well-watered treatment (WW) of Neumann et al. (2017). The lighter blue color indicates a non-significant correlation.

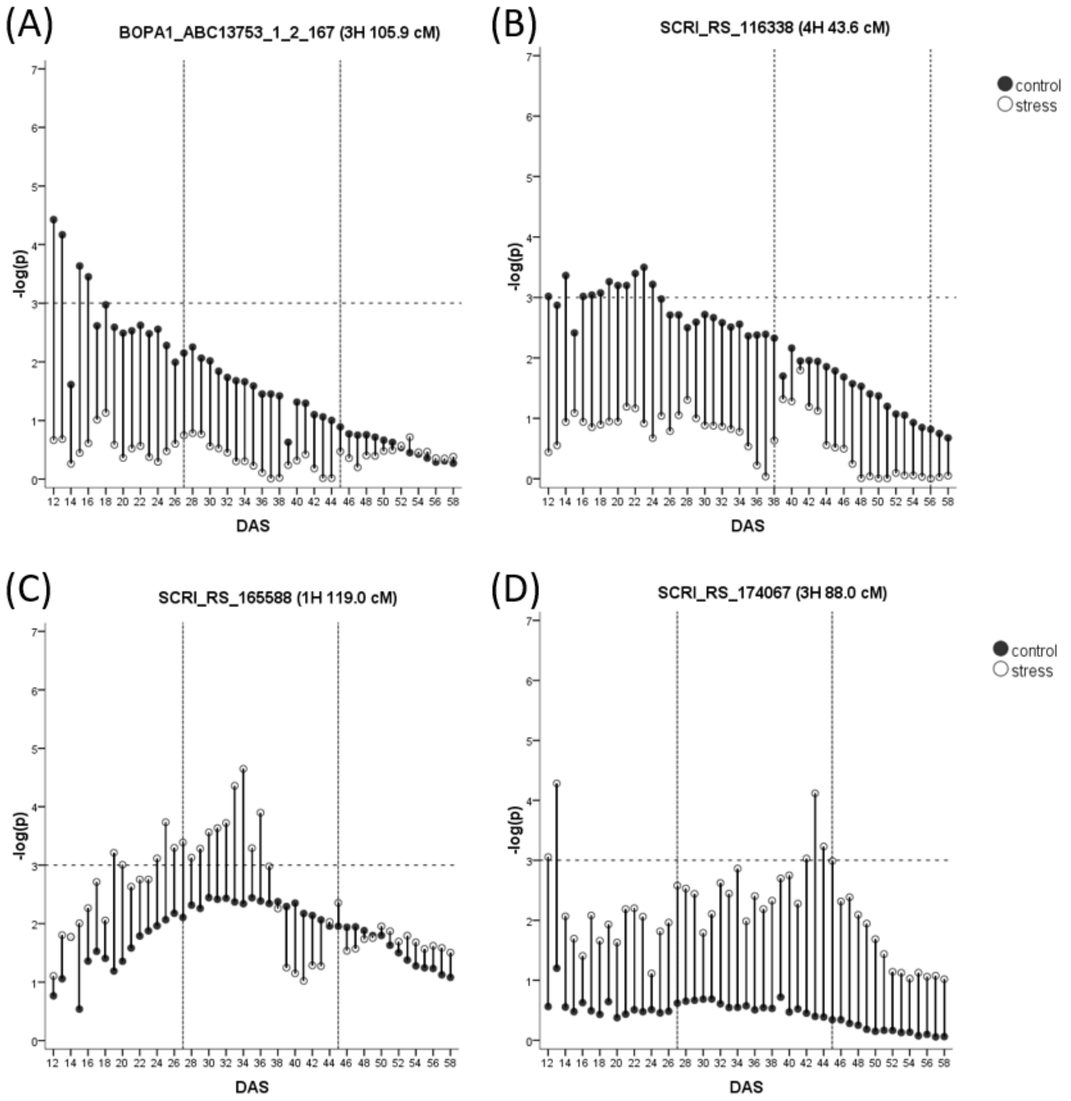

Fig. S17 A-D Adaptive biomass QTL with their –log(p)-values over time in days after sowing (DAS) in drought stress (unfilled) and well-watered (black) conditions. Presented is only the most significant SNP of each QTL (in case it consists of several SNPs). As the FDR is calculated for each day and in each treatment separately, the general significance level of –log(p)-value = 3 is indicated by a dashed horizontal line. The drought stress phase from DAS 27 to DAS 45 is indicated by the two dashed vertical lines.
